# A mosquito salivary protein promotes flavivirus transmission by activation of autophagy

**DOI:** 10.1038/s41467-019-14115-z

**Published:** 2020-01-14

**Authors:** Peng Sun, Kaixiao Nie, Yibin Zhu, Yang Liu, Pa Wu, Ziwen Liu, Senyan Du, Huahao Fan, Chun-Hong Chen, Renli Zhang, Penghua Wang, Gong Cheng

**Affiliations:** 1 Tsinghua-Peking Center for Life Sciences, School of Medicine, Tsinghua University, Beijing, 100084 China; 2 Institute of Pathogenic Organisms, Shenzhen Center for Disease Control and Prevention, Shenzhen, Guangdong 518055 China; 3 School of Life Sciences, Tsinghua University, Beijing, 100084 China; 4 National Institute of Infectious Diseases and Vaccinology, National Health Research Institutes, Zhunan, Miaoli, Taiwan 35053 China; 5 Department of Immunology, School of Medicine, The University of Connecticut Health Center, Farmington, Connecticut 06030 USA

**Keywords:** Autophagy, Pathogens, Virology

## Abstract

Transmission from an infected mosquito to a host is an essential process in the life cycle of mosquito-borne flaviviruses. Numerous studies have demonstrated that mosquito saliva facilitates viral transmission. Here we find that a saliva-specific protein, named *Aedes aegypti* venom allergen-1 (*Aa*VA-1), promotes dengue and Zika virus transmission by activating autophagy in host immune cells of the monocyte lineage. The AG6 mice (*ifnar1*^–/–^*ifngr1*^–/–^) bitten by the virus-infected *Aa*VA-1-deficient mosquitoes present a lower viremia and prolonged survival. *Aa*VA-1 intracellularly interacts with a dominant negative binder of Beclin-1, known as leucine-rich pentatricopeptide repeat-containing protein (LRPPRC), and releases Beclin-1 from LRPPRC-mediated sequestration, thereby enabling the initialization of downstream autophagic signaling. A deficiency in Beclin-1 reduces viral infection in mice and abolishes *Aa*VA-1-mediated enhancement of ZIKV transmission by mosquitoes. Our study provides a mechanistic insight into saliva-aided viral transmission and could offer a potential prophylactic target for reducing flavivirus transmission.

## Abstract (web summary)

Mosquito saliva affects transmission of flaviviruses, but underlying mechanisms are incompletely understood. Here, the authors show that *Aedes aegypti* venom allergen-1 (*Aa*VA-1) promotes dengue and Zika virus transmission by activating autophagy in host immune cells of the monocyte lineage.

## Introduction

Flavivirus is a genus of single-stranded RNA viruses that are often transmitted by arthropod vectors, such as ticks and mosquitoes. Mosquito-borne flaviviruses, including dengue (DENV), Zika (ZIKV), yellow fever, and West Nile (WNV) viruses, cause hundreds of millions of infection cases annually and act as the etiological agents of human hemorrhagic fever, encephalitis, and meningitis^1^. DENV, which is transmitted by *Aedes aegypti*, is the widest spreading arbovirus worldwide^2^. There are more than 100 dengue-endemic countries in the world; ~390 million people are infected by DENV every year, mostly in tropical countries^2^. ZIKV is another mosquito-borne flavivirus and is transmitted to humans by *Aedes* mosquito species^3^. Several neurological complications, such as Guillain-Barré syndrome in adults and microcephaly in neonates, are potentially associated with ZIKV infection^4–6^. Mosquito-borne flaviviruses maintain a life cycle between mosquitoes and susceptible hosts in which viral transmission from an infected mosquito to a host is an essential process for viral survival in nature^7^. Through viral transmission, the infectious virions are injected into the host dermis by a mosquito bite^8^ and then robustly replicate in dermal-residing monocyte-lineage immune cells^9–11^, thereby establishing the initial infection in hosts. Subsequently, the viruses are released from the infected immune cells into the blood circulation for systemic dissemination in hosts^12^.

Mosquito saliva, containing proteins with angiogenic, antihemostatic, anti-inflammatory, and immunomodulatory properties, is inoculated together with viruses into the host during viral transmission^13,14^. Numerous studies have demonstrated that mosquito saliva can facilitate viral transmission and contribute to the subsequent disease sequelae. For example, inoculation of WNV together with salivary gland extract (SGE) results in higher viremia and faster neuroinvasion compared with WNV inoculation alone via needles^15^. Mice bitten by infected mosquitoes develop higher and sustained DENV viremia compared with those infected by direct needle injection^16^, suggesting that the salivary proteins promote flavivirus transmission and pathogenesis in bitten hosts. Nonetheless, the underlying mechanisms of salivary proteins in flaviviral transmission remain to be understood.

Autophagy is an evolutionarily conserved stress-responsive cytosolic process that disposes of unnecessary or dysfunctional cellular components^17^. In mammals, autophagy initiation starts with the activation of the ULK1 (unc-51 like autophagy activating kinase 1) complex^18^. The ULK1 complex consists of ULK1 itself and the non-catalytic subunits FIP200 (RB1 inducible coiled-coil 1), ATG13 (autophagy-related 13), and ATG101. The ULK1–ATG13–FIP200–ATG101 complex is present mainly in the cytosol under nutrient-rich conditions and is inactivated by mTORC1 (mammalian target of rapamycin complex 1)^19^. Occurring just downstream of ULK1 activation, phosphatidylinositol 3-kinase (PI3KC3) class III phosphorylates the lipid head group of phosphatidylinositol to generate phosphatidylinositol 3-phosphate, which is essential for canonical autophagosome formation. PI3KC3 forms at least two distinct complexes known as complex I and II (PI3KC3-C1 and PI3KC3-C2)^20^. Both complexes contain VPS34 (PI3KC3 catalytic subunit type 3), VPS15 (phosphoinositide-3-kinase regulatory subunit 4), and Beclin-1. PI3KC3-C1 contains ATG14, which directs the complex to phagophore initiation sites to facilitate elongation^20^. PI3KC3-C2 contains UVRAG (UV radiation resistance-associated gene), which directs endosome and autophagosome maturation^21^. The autophagosome is delivered to lysosomes for degradation.

In this study, we screen the roles of *A. aegypti* salivary proteins during DENV and ZIKV infection of human immune cells, and find that *A. aegypti* venom allergen-1 (*Aa*VA-1) acts as a female mosquito saliva-specific protein to promote flaviviral transmission through the activation of host autophagy, which promotes infections by many viruses in the *Flaviviridae* family^22–24^. Our mechanistic study indicates that *Aa*VA-1 competes with an autophagy inhibitor, leucine-rich pentatricopeptide repeat (PPR)-containing protein (LRPPRC), thereby enabling the activation of autophagic signaling.

## Results

### *Aa*VA-1 promotes flavivirus infection in human immune cells

**Fig. 1:**
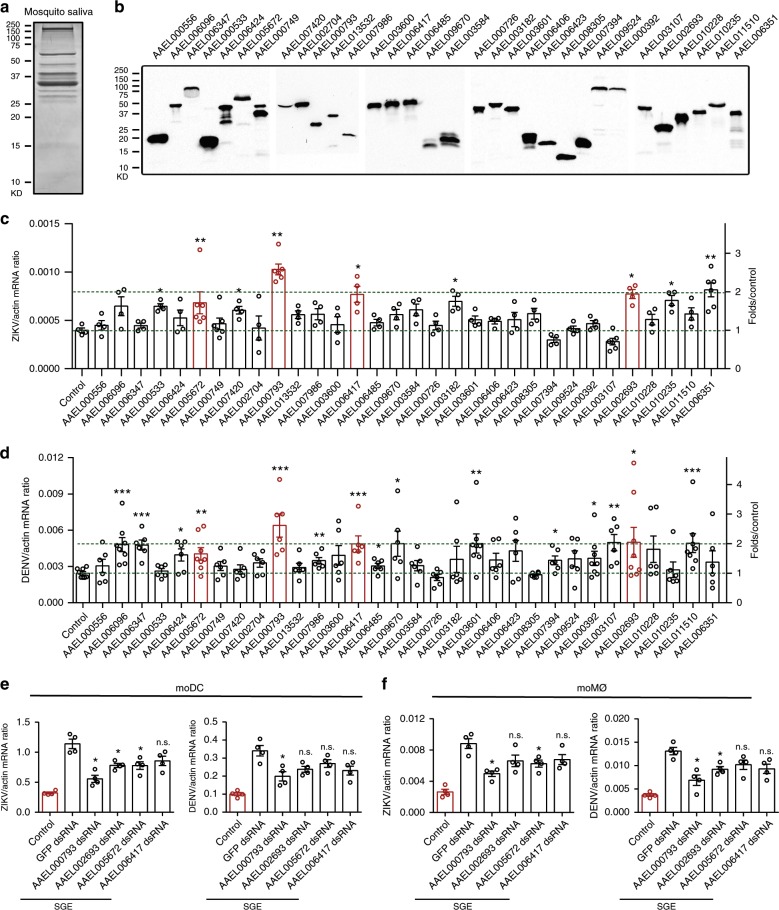
Identification of *Aa*VA-1 from mosquito saliva to facilitate flavivirus infection. **a** Separation of *A. aegypti* saliva by SDS–PAGE. The saliva was collected from 2000 female *A. aegypti* by sucrose meals using an in vitro membrane feeding system. The sucrose buffer with *A. aegypti* saliva was concentrated by lyophilization and then resuspended in PBS for separation by SDS–PAGE and stained with Coomassie Blue. **b** Expression of *A. aegypti* salivary proteins in *Drosophila* S2 cells. Thirty-two genes with a high mass spectrometry score (>25) were cloned into the pMT/BiP/V5-His A expression vector and expressed in *Drosophila* S2 cells. Expression was detected by western blotting with anti-V5 antibody. The experiments were repeated two times with the similar results. **c**, **d** The role of *A. aegypti* salivary proteins during ZIKV (**c**) and DENV (**d**) infection of human THP-1 cells. Conditioned supernatants with recombinant salivary proteins were mixed with either ZIKV (0.1 multiplicity of infection (MOI)) or DENV (0.1 MOI) to infect the human monocytic cell line THP-1. Mock supernatant served as a control. The infected cells were collected 24 h post infection for detection of viral genomes by qRT-PCR. **c** *n* = 4 or *n* = 6 independent samples. **d** *n* = 6 or *n* = 8 independent samples. **e**, **f** Depletion of salivary proteins impaired the SGE-mediated enhancement of both DENV and ZIKV infection in human moDC (**e**) and moMØ (**f**). Either 0.5 MOI or 0.1 MOI viruses were used to infect moDC or moMØ, respectively. *n* = 4 independent samples. **c**–**f** Gene quantities were normalized against human *actin* (NM_001101.4). The data are presented as the mean ± SEM. A nonparametric Mann–Whitney test was used for the statistical analysis. *p *<* 0.05, **p *<* 0.01, ***p *<* 0.001, n.s., not significant (*p* ≥ 0.05). The data were combined based on two independent experiments (**c**, **e**, **f**) or three independent experiments (**d**). Source data are provided as a Source Data file.

Mosquito salivary proteins are intradermally inoculated with viruses into a host simultaneously through a mosquito bite. However, the roles of mosquito salivary proteins in flavivirus infection remain to be comprehensively investigated. Therefore, we collected *A. aegypti* saliva by sucrose meals with an in vitro membrane feeding system^25^ and then identified the proteins by SDS–polyacrylamide gel electrophoresis (PAGE) and mass spectrometry (Fig. 1a). Seventy-one proteins were identified from the *A. aegypti* saliva (Supplementary Table 1). Subsequently, 42 genes with the score more than 25 in mass spectrometry were selected (Supplementary Table 1), in which 32 genes were successfully cloned and expressed in *Drosophila* S2 cells (Fig. 1b). The conditioned supernatants with recombinant salivary proteins were mixed with either ZIKV or DENV to infect a human monocytic cell line THP-1. Notably, incubation of salivary proteins encoded by *AAEL000793*, *AAEL002693*, *AAEL005672*, and *AAEL006417* genes resulted in a robust replication (*p* *<* 0.05) of both ZIKV (Fig. 1c) and DENV (Fig. 1d) in THP-1 cells, suggesting a susceptibility role of these proteins in viral transmission.

The viruses inoculated by mosquitoes primarily infect dermal-resident monocyte-lineage cells^8,9,11^. The viruses are then released from the primary infected cells into the blood circulation, thereby causing subsequent viremia and diseases (Supplementary Fig. 1A). We next examined whether these salivary proteins could regulate viral replication in dendritic cells (moDC) and macrophages (moMØ) derived from human monocytes^26,27^. The identified salivary genes (*AAEL000793*, *AAEL005672*, *AAEL006417*, and *AAEL002693*) were silenced in *A. aegypti* by double-stranded RNA (dsRNA) thoracic inoculation (Supplementary Fig. 1B–E). The SGE of mock-treated mosquitoes facilitated DENV and ZIKV infection in both moDC and moMØ, while silencing these genes impaired the SGE-mediated enhancement of both DENV and ZIKV infection. Knockdown of *AAEL000793* resulted in the greatest reduction (Fig. 1e, f). *AAEL000793* is a member of a multigene family annotated as venom allergen. There are multiple venom allergen paralogs in *A. aegypti* identified by sequence comparison (Supplementary Table 2). Notably, incubation of a protein encoded by *AAEL002693*, a paralog of *AAEL000793* (42% amino acid identity), also facilitated infection by ZIKV and DENV (Fig. 1c–f), further suggesting the pro-viral role of the mosquito salivary venom allergen during flaviviral transmission^28^. As genetic manipulation of *AAEL000793* consistently presented the most dramatic phenotype among all these salivary genes, we selected *AAEL000793* for further investigation and therefore designated *AAEL000793* as *Aa*VA-1 throughout this study.

**Fig. 2:**
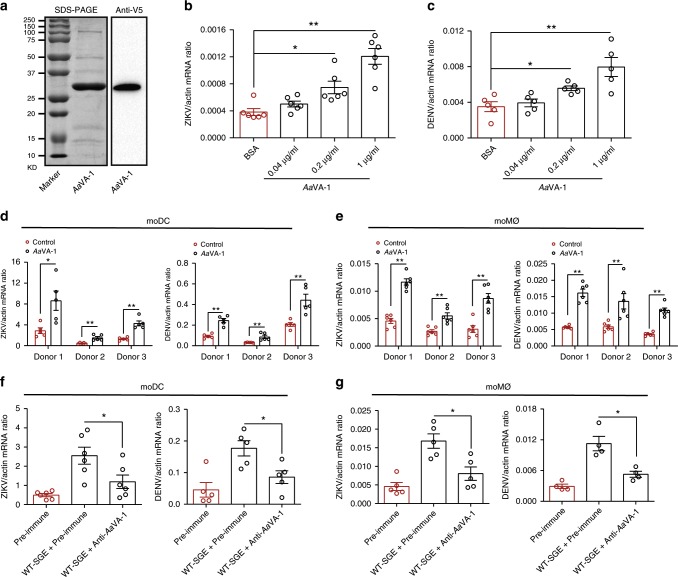
*Aa*VA-1 promotes flaviviral infection in human monocyte-lineage cells. **a** Expression and purification of *Aa*VA-1 using a *Drosophila* expression system. The *Aa*VA-1 gene was cloned into the pMT/BiP/V5-His A expression vector. Recombinant *Aa*VA-1 was expressed and purified using a Cobalt-His column (left panel). Expression was probed using anti-V5 antibody (right panel). The experiments were repeated three times with the similar results. **b**, **c** Incubation of *Aa*VA-1 purified protein facilitated ZIKV (**b**) and DENV (**c**) infection in THP-1 cells. The infected cells were detected at 24 h postinfection by qRT-PCR. BSA was incubated with the viruses as a mock control. **b** *n* = 6 independent samples. **c** *n* = 5 independent samples. **d**, **e** Incubation of *Aa*VA-1 enhanced the replication of ZIKV and DENV in human moDC (**d**) and moMØ (**e**). The same amount of BSA served as a negative control. **d** *n* = 5 independent samples. **e** *n* = 6 independent samples. **f**, **g** Immuno-blockade of *Aa*VA-1 by a murine polyclonal antibody restored the WT-SGE-mediated viral enhancement in human moDC (**f**) and moMØ (**g**). Pre-immune sera mixed with SGE served as a mock control. **f** *n* = 6 (ZIKV) or *n* = 5 (DENV) independent samples. **g** *n* = 5 (ZIKV) or *n* = 4 (DENV) independent samples. **b**–**g** Human cells were infected with ZIKV and DENV, respectively. The viral doses used in the infection were 0.1 MOI for THP-1, 0.5 MOI for moDC, and 0.1 MOI for moMØ. Gene quantities were normalized against human *actin* (NM_001101.4). The data are presented as the mean ± SEM. A nonparametric Mann–Whitney test was used for the statistical analysis. *p < 0.05, **p < 0.01. The data were combined based on two independent experiments. Source data are provided as a Source Data file.

**Fig. 3:**
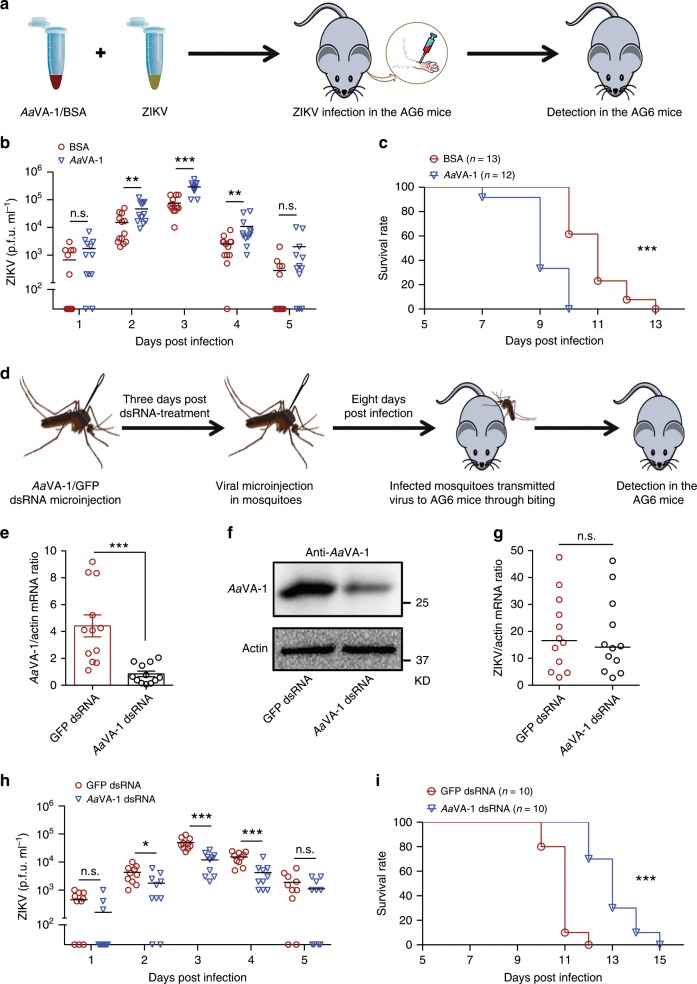
*Aa*VA-1 promotes flaviviral transmission via biting by *A. aegypti*. **a**–**c** Inoculation of purified *Aa*VA-1 facilitated ZIKV infection in AG6 mice. **a** Schematic representation of the study design. Purified *Aa*VA-1 (2 μg per mouse) with ZIKV (500 p.f.u.) was subcutaneously inoculated into the footpads of AG6 mice. The same amount of BSA with ZIKV served as mocks. **b** The ZIKV load in mouse plasma was detected using a plaque assay. **c** Animal mortality was recorded daily (*n* = 13 (BSA) or *n* = 12 (*Aa*VA-1) mice per group). **d**–**i** Knockdown of *Aa*VA-1 in *A. aegypti* impaired ZIKV transmission to the host. **d** Schematic representation of the study design. The *AaVA-1* gene was silenced by dsRNA in *A. aegypti*. The mosquitoes inoculated with *GFP* dsRNA served as mocks. After 3 days, the dsRNA-treated mosquitoes were thoracically infected with ZIKV. After rearing 8 days, the infected mosquitoes bit AG6 mice. Mouse viremia and survival were recorded daily. **e**, **f** The *AaVA-1* silencing efficiency in *A. aegypti* salivary glands was detected by qRT-PCR (**e**) and western blotting (**f**) at 11 days post gene silencing. **e** The data are presented as the mean ± SEM. **f** The experiments were repeated three times with the similar results. **g** The ZIKV burden was determined at 8 days postinfection. **e**, **g** Gene quantities were normalized against *A. aegypti actin* (*AAEL011197*). *n* = 12 mosquito salivary glands per group. **h** The ZIKV load in mouse plasma was detected using a plaque assay. **i** Animal mortality were recorded daily (*n* = 10 mice per group). **b**, **e**, **g**, **h** A nonparametric Mann–Whitney test was used for the statistical analysis. Each line represents the median value (**g**) or mean value (**b**, **h**) of the group. **c**, **i** Survival rates of the infected mice were statistically analysed using the log-rank (Mantel–Cox) test. *p < 0.05, **p < 0.01, ***p < 0.001, n.s., not significant (*p* ≥ 0.05). The data were combined based on three independent experiments (**b**, **c**) or 2 independent experiments (**e**, **g**, **h**, **i**). Source data are provided as a Source Data file.

To further validate the role of *Aa*VA-1 in these flaviviral infections, we expressed and purified *Aa*VA-1 in *Drosophila* S2 cells (Fig. 2a). The purified *Aa*VA-1 enhanced the replication of ZIKV (Fig. 2b) and DENV (Fig. 2c) in THP-1 cells in a dose-dependent manner. Intriguingly, *Aa*VA-1 was specifically expressed in the salivary glands of female *A. aegypti* (Supplementary Fig. 2A) rather than male mosquitoes (Supplementary Fig. 2B). *Aa*VA-1 was not induced by DENV and ZIKV infections in mosquito salivary glands (Supplementary Fig. 3A–C). We further detected *Aa*VA-1 expression in the salivary glands of female mosquitoes 4 days after a blood meal. Compared with that of sugar-fed mosquitoes, the expression of *Aa*VA-1 did not change in the salivary glands of blood-fed mosquitoes (Supplementary Fig. 3D). We next examined whether *Aa*VA-1 could enhance viral replication in moDC and moMØ derived from human monocytes^26,27^. Incubation of *Aa*VA-1 enhanced the replication of ZIKV and DENV in human moDC (Fig. 2d) and moMØ (Fig. 2e). Consistently, the SGE of wild-type *A. aegypti* (WT-SGE) facilitated DENV and ZIKV infection in both moDC (Fig. 3f) and moMØ (Fig. 3g), whereas immuno-blockade of *Aa*VA-1 by a murine polyclonal antibody (Supplementary Fig. 4) impaired the SGE-mediated viral enhancement (Fig. 3f, g), further validating susceptibility pro-viral role of *Aa*VA-1 in flaviviral infection of human immune cells. Given the genetic variations among human individuals, we next assessed the role of *Aa*VA-1 in flavivirus infection of immune cells from different individual donors. *Aa*VA-1 universally enhanced both DENV (Supplementary Fig. 5A) and ZIKV (Supplementary Fig. 5B) infections in monocytes from several individuals. Taken together, *Aa*VA-1 is a mosquito salivary protein that promotes flaviviral replication in human monocyte-lineage immune cells.

### *Aa*VA-1 facilitates flaviviral transmission to mice

We next assessed the role of *Aa*VA-1 in flaviviral transmission in a type I/II interferon receptor-deficient (*ifnar1*^*–/–*^*ifngr1*^*–/–*^) C57BL/6 (AG6) mouse model that was established in previous studies^29^. First, we subcutaneously inoculated the purified *Aa*VA-1 with ZIKV into the footpads of AG6 mice (Fig. 3a). Inoculation of *Aa*VA-1 augmented ZIKV viremia (Fig. 3b) and accelerated animal death (Fig. 3c), indicating that *Aa*VA-1 is a salivary factor promoting ZIKV infection and pathogenesis in the host. Next, we assessed the role of *Aa*VA-1 in viral transmission using a “mosquito-AG6 mouse” transmission model (Fig. 3d). The *AaVA-1* gene was silenced by dsRNA thoracic microinjection in *A. aegypti* salivary glands (Fig. 3e, f). Three days post gene silencing, the dsRNA-treated mosquitoes were thoracically infected with ZIKV. These infected mosquitoes were allowed to bite AG6 mice. Mouse viremia and survival were subsequently recorded every day after mosquito biting (Fig. 3d). Silencing of the *AaVA-1* gene did not influence the ZIKV load in the mosquito salivary glands (Fig. 3g). Notably, AG6 mice that were bitten by the *AaVA-1*-silenced ZIKV-infected mosquitoes had lower viremia (Fig. 3h) and survived longer (Fig. 3i) than the animals bitten by control mosquitoes (*GFP* dsRNA), indicating that *Aa*VA-1 plays an important role in flavivirus transmission by mosquitoes.

Next, we identified a homolog of *AaVA-1* with the highest identity in the *Aedes albopictus* genome (*AalbVA-1*) (Supplementary Fig. 6A). The recombinant *Aalb*VA-1 was expressed and purified in *Drosophila* S2 cells (Supplementary Fig. 6B). Incubation of *Aalb*VA-1 enhanced infection by ZIKV in both moDC (Supplementary Fig. 6c) and moMØ (Supplementary Fig. 6D) from different human donors. We next validated the role of *Aalb*VA-1 in flaviviral transmission in a mosquito-biting model. Knockdown of *AalbVA-1* (Supplementary Fig. 6E, F) did not alter ZIKV replication in the *A. albopictus* salivary glands (Supplementary Fig. 6G); however, *Aalb*VA-1 deficiency in mosquito saliva impaired ZIKV viremia in mice (Supplementary Fig. 6H) and delayed animal death (Supplementary Fig. 6I). Taken together, the *Aedes* VA-1 protein generally plays a pro-viral role in flaviviral transmission.

### *Aa*VA-1 activates autophagy to promote flaviviral infection

Mosquito saliva is able to modulate the host inflammatory immune response^12,28,30,31^. Therefore, we assessed *Aa*VA-1-mediated cytokine regulation. Incubation of *Aa*VA-1 in THP-1 cells did not influence the expression of tumor necrosis factor-α (TNF-α), interleukin-1β (IL-1β), IL-8, monocyte chemotactic protein-1 (MCP-1), interferon-beta (IFN-β), interferon-alpha 2 (IFN-α2), IFN-α7 or macrophage inflammatory protein-1α (MIP-1α) (Supplementary Fig. 7), indicating that the *Aa*VA-1-mediated enhancement of infection may not be attributed to the regulation of host immune responses. In addition, *Aa*VA-1 did not directly interact with the purified envelope protein of DENV (Supplementary Fig. 8).

**Fig. 4:**
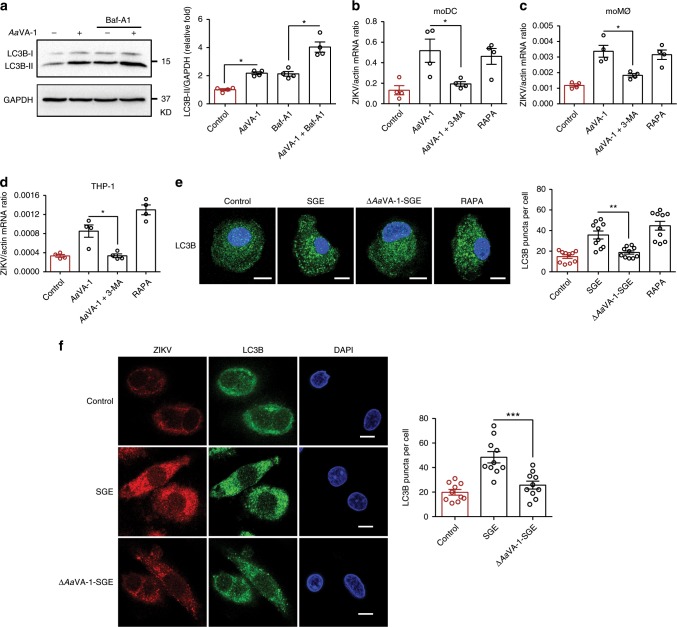
*Aa*VA-1 facilitates flaviviral infection by activating autophagy. **a** The role of *Aa*VA-1 in activation of autophagy in human THP-1 cells. *Aa*VA-1 (1 μg/ml) was incubated with THP-1 cells with or without Baf-A1. An equal amount of BSA served as a mock control. At 6 h post incubation, autophagy activity was assessed by western blotting with anti-LC3B antibody (left panel), and quantification of the signaling intensity of LC3B-II relative to GAPDH (right panel). *n* = 4 independent experiments. **b**–**d** Interruption of autophagy with the antagonist 3-MA impaired *Aa*VA-1-mediated ZIKV enhancement in moDC (**b**), moMØ (**c**), and THP-1 cells (**d**). *Aa*VA-1 (1 μg/ml) was incubated with the cells with or without 5 mM 3-MA. An equal amount of BSA served as a mock control. The autophagy agonist RAPA was used as a positive control. At 24 h post incubation, the viral load was detected by qRT-PCR. Gene quantities were normalized against human *actin* (NM_001101.4). *n* = 4 independent samples. The experiments were repeated three times with the similar results. **e**, **f** *Aa*VA-1 in the *A. aegypti* SGE enhanced both antophagic activation and ZIKV replication in human moMØ. Human moMØ cells were infected by ZIKV (0.1 MOI) in combination with either Δ*Aa*VA-1-SGE or WT-SGE. **e** After 6 h infection, the cells were stained with LC3B (Green) antibody (left panel). RAPA served as a positive control. LC3B puncta were quantified in the right panel. **f** After 48 h infection, the cells were then stained with both LC3B (Green) and flavivirus E protein (4G2) (Red) antibodies. LC3B puncta were quantified in the right panel. The nuclei were stained with DAPI. Images were examined by Zeiss LSM 780 meta confocal microscopy. Scale bars, 10 μm. *n* = 10 cells per group. The experiments were repeated three times with the similar results. **a**–**f** The data are presented as the mean ± SEM. A nonparametric Mann–Whitney test was used for the statistical analysis. *p < 0.05, **p < 0.01, ***p < 0.001. Source data are provided as a Source Data file.

Autophagy is a well-known cell-intrinsic pathway that regulates flaviviral infection. A deficiency in the autophagic pathway interrupts the replication of DENV^22^, ZIKV^24^, Japanese encephalitis virus (JEV)^32^ and hepatitis C virus (HCV)^33^ in human cells. We next assessed the role of *Aa*VA-1 in the activation of autophagy in human monocyte-lineage immune cells. *Aa*VA-1 induced the conversion of LC3B-I into LC3B-II in THP-1 cells (Fig. 4a) and the accumulation of LC3B-positive puncta in human moDC and moMØ (Supplementary Fig. 9A). Indeed, enhancement of LC3B-II and LC3B-positive puncta formation may be attributed to either activation of autophagy or decreased degradation of autophagic membrane stabilization^34^. Bafilomycin A1 (Baf-A1) is a known inhibitor of the late phase of autophagy that prevents the degradation of autophagic vacuoles by inhibiting fusion between autophagosomes and lysosomes^35^. Treatment with Baf-A1 increased the accumulation of LC3B-II and LC3B-positive puncta upon *Aa*VA-1 exposure (Fig. 4a and Supplementary Fig. 9B), suggesting that *Aa*Va-1-mediated activation of autophagy contributed to the enhancement of autophagy rather than the impairment of lysosome-mediated autophagosome degradation. 3-Methyladenine (3-MA) is an autophagy antagonist to inhibit autophagic signaling by blocking the VPS34/PI3 kinase complex^24^. Consistently, the interruption of autophagy by 3-MA impaired the *Aa*VA-1-mediated ZIKV enhancement in moDC (Fig. 4b), moMØ (Fig. 4c), and THP-1 cells (Fig. 4d). Conversely, an autophagy inducer, rapamycin (RAPA)^24^ as a positive control, increased ZIKV replication in these cells. *Aa*VA-1 presented a similar role in DENV infection of these human immune cells (Supplementary Fig. 9C–E). To mimic the physiological conditions, human moMØ cells were incubated with either Δ*Aa*VA-1-SGE or WT-SGE for immunofluorescence staining. *Aa*VA-1-knockdown SGE failed to induce autophagy and ZIKV replication as WT-SGE did in the primary immune cells (Fig. 4e-f), demonstrating that the autophagy-inducing and pro-viral feature of *Aa*VA-1 is common to DENV and ZIKV. Autophagy has been described to restrict cytosolic DNA and RNA sensors^36^. We therefore assessed the role of *Aa*VA-1 in infection of other arboviruses. *Aa*VA-1 did not regulate Batai virus (*Orthobunyavirus*, *Bunyaviridae* family) infection (Supplementary Fig. 10A); however, *Aa*VA-1 enhanced the Semliki Forest virus (*Alphavirus*, *Togaviridae* family) burden in human immune cells (Supplementary Fig. 10B), suggesting that either *Aa*VA-1 or *Aa*VA-1-mediated autophagic signaling may play distinct roles in the infection of arboviruses from different families^37^.

**Fig. 5:**
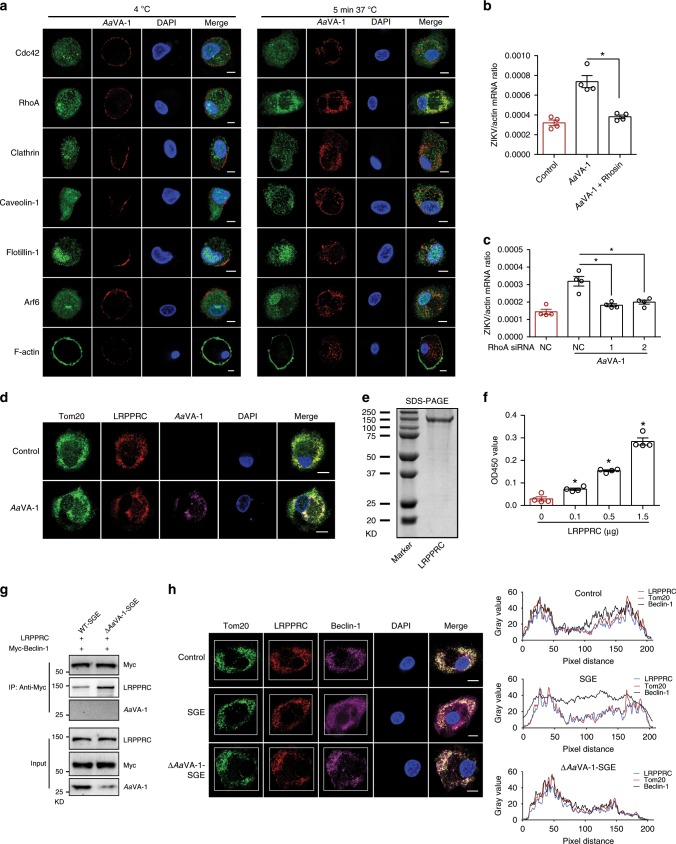
*Aa*VA-1 competes with Beclin-1 from the LRPPRC-mediated inhibition. **a** The localization of *Aa*VA-1 in human moMØ cells. Purified *Aa*VA-1 (5 μg/ml) was incubated with human moMØ cells at 4 °C or 37 °C. Both *Aa*VA-1 and specific markers of the endocytosis pathways were stained, and nuclei were stained with DAPI. Scale bars, 10 μm. **b**, **c** Suppression of RhoA by the antagonist Rhosin (**b**) and siRNA-mediated silencing (**c**) restored *Aa*VA-1-mediated ZIKV enhancement in THP-1 cells. Gene quantities were normalized against human *actin* (NM_001101.4). **d** Colocalization of *Aa*VA-1 and LRPPRC on mitochondria in SGE-treated human moMØ cells. Purified *Aa*VA-1 (biotin-labeled, 5 μg/ml) was incubated with human moMØ for 6 h. The cells were stained with anti-LRPPRC and anti-Tom20, as well as streptavidin Alexa-633. The nuclei were stained with DAPI. Images were examined using a Zeiss LSM 780 meta confocal microscope in multi-track mode. Scale bars, 10 μm. **e** Purification of a recombinant LRPPRC protein in 293F cells. The protein was detected by staining with Coomassie blue in an SDS–PAGE gel. **f** *Aa*VA-1 directly binds LRPPRC. This interaction was detected by ELISA. **g** *Aa*VA-1 impaired the binding between LRPPRC and Beclin-1. SGE or Δ*Aa*VA-1-SGE was incubated with the lysates from Beclin-1 (Myc tag)-expressing cells to investigate protein interactions. The protein complex was pulled down with anti-Myc antibodies. **h** Treatment of SGE dissociated Beclin-1 from LRPPRC on mitochondria. The human moMØ cells were infected ZIKV (0.1 MOI) in combination with either Δ*Aa*VA-1-SGE or WT-SGE. After 6 h of incubation, the cells were stained with anti-Beclin-1, anti-LRPPRC, and anti-Tom20. The nuclei were stained with DAPI. Images were examined using a Zeiss LSM 780 meta confocal microscope. Scale bars, 10 μm. The colocalization of Beclin-1 and LRPPRC on mitochondria was quantified in the white rectangle (right panel). **b**, **c**, **f** The data are presented as the mean ± SEM. A nonparametric Mann–Whitney test was used for the statistical analysis. *p < 0.05. *n* = 4 independent samples. The experiments were repeated two times (**a**, **e**–**g**) or three times (**b**–**d**, **h**) with the similar results. Source data are provided as a Source Data file.

To investigate the mechanism by which *Aa*VA-1 starts autophagy, we assessed the location of *Aa*VA-1 in human immune cells. Either human moDC or moMØ cells were incubated with the purified *Aa*VA-1 to detect the cellular distribution of *Aa*VA-1 through an immunofluorescence assay. Intriguingly, *Aa*VA-1 was found in the cytoplasm of human immune cells (Supplementary Fig. 11A), suggesting that *Aa*VA-1 may be taken up by immune cells. We next investigated how *Aa*VA-1, an external protein from mosquito saliva, gains access into human cells. *Aa*VA-1 is most likely delivered into human immune cells by endocytosis. Endocytic pathways are classified into three types by their dependence on the lipid rafts. (i) Pathways that do not involve lipid rafts in the endocytic vesicle, namely clathrin-mediated endocytosis (CME). (ii) Endocytic pathways for which the endocytic vesicle contains lipid rafts together with non-raft membrane domains, such as phagocytosis. (iii) Endocytic pathways taking place in lipid rafts in a clathrin-independent manner, such as caveolae-mediated, flotillin-dependent, Cdc42-dependent, Arf6-dependent, and RhoA-dependent endocytosis^38^. The staining showed that *Aa*VA-1 attached to the surface of the human macrophages at 4 °C incubation (Fig. 5a). Nonetheless, *Aa*VA-1 was quickly internalized into cells, and colocalized with RhoA after 5 min of incubation at 37 °C (Fig. 5a). Indeed, functional blockade of RhoA by its inhibitor Rhosin^39^ suppressed *Aa*VA-1-mediated enhancement of ZIKV infection in THP-1 cells (Fig. 5b). Consistent with this result, small interfering RNA (siRNA)-mediated silencing of *RhoA* reduced ZIKV infection in the *Aa*VA-1 incubated THP-1 cells (Fig. 5c and Supplementary Fig. 11B). Altogether, *Aa*VA-1 is endocytosed into human immune cells in a RhoA-dependent manner.

We next investigated the intracellular localization of *Aa*VA-1 after endocytosis. In this experiment, the human monocyte-derived macrophages were incubated with *Aa*VA-1 and then the cells were collected to stain cell organelles, such as early endosome (Rab5), late endosome (Rab7), Golgi (GM130), endoplasmic reticulum (Calnexin), lysosome (Lamp1), and mitochondria (Tom20), in a time course post incubation. *Aa*VA-1 was colocalized with the early and late endosomes at 10 and 30 min post incubation (Supplementary Fig. 11C). *Aa*VA-1 colocalized with Tom20 at 6 h post incubation, suggesting that the intracellular trafficking route of *Aa*VA-1 is to escape from endosome to mitochondria (Supplementary Fig. 11C).

### *Aa*VA-1 liberates Beclin-1 from the LRPPRC-bound state

Given that *Aa*VA-1 plays a role in flaviviral infection of THP-1 cells, we next identified the *Aa*VA-1-binding proteins in THP-1 cell lysates through an immuno-pulldown assay (Supplementary Fig. 12A). The bound proteins were separated by SDS–PAGE and subsequently characterized by a liquid chromatograph-mass spectrophotometry assay (LC-MS). Overlapping proteins pulled down also in the control were excluded. The *Aa*VA-1-binding proteins with a score >30 in the LC-MS assay were further investigated (Supplementary Table 3). Intriguingly, an autophagy inhibitor, named LRPPRC, was pulled down by *Aa*VA-1 (Supplementary Fig. 12B). Previous studies indicated that LRPPRC acts as a Beclin-1 suppressor through directly interacting with and preventing Beclin-1 from PI3KCIII-mediated autophagy initiation^40–42^. The interaction between *Aa*VA-1 and LRPPRC was validated by co-immunoprecipitation (co-IP) (Supplementary Fig. 12C). We further validated colocalization between endogenous *Aa*VA-1 of *A. aegypti* SGE with endogenous LRPPRC in human primary moMØ cells (Supplementary Fig. 12D). LRPPRC is mainly located on mitochondria^43^. Confocal microscopy demonstrated that a high level of LRPPRC was present on mitochondria and a substantial amount of *Aa*VA-1 co-stained with LRPPRC and a mitochondrial marker (Tom20) at 6 h post incubation (Fig. 5d). We next expressed and purified the LRPPRC recombinant protein in human 293F cells (Fig. 5e). *Aa*VA-1 interacted with LRPPRC in a dose-dependent manner (Fig. 5f). To further determine the binding regions of LRPPRC to *Aa*VA-1, we next constructed four truncations of LRPPRC (T1: 1–337 aa; T2: 338–711 aa; T3: 712–1067 aa; T4: 1068–1394 aa). The LRPPRC-T3 fragment (712–1067 aa) was responsible for the interaction with *Aa*VA-1 (Supplementary Fig. 12E). In addition, *Aa*VA-1 directly interacted with the purified LRPPRC-T3 fragment (Supplementary Fig. 12F–G). Altogether, our data suggest that *Aa*VA-1 directly binds to LRPPRC on the mitochondria.

LRPPRC is a member of PPR protein family^44^. The PPR proteins constitute a large family of RNA-binding proteins that contain a canonical 35 residue repeat motif^45^. There are seven proteins with PPR domains in human (Supplementary Fig. 13A)^44^. To further investigate the binding specificity between *Aa*VAs and human PPRs, we next selected a paralogue of the *Aa*VA gene, *Aa*VA-9 (*AAEL009695*), an *Aa*VA subtype predominantly expressed in the mosquito head and hemolymph (Supplementary Fig. 13B), and a member of the PPR family, named PTCD1 (PPR-containing protein-1), for the investigation. *Aa*VA-1 and *Aa*VA-9 were expressed in *Drosophila* S2 cells, while human LRPPRC and PTCD1 were ectopically generated in human 293T cells. LRPPRC but not PTCD1 efficiently pulled down *Aa*VA-1 (Supplementary Fig. 13C). Consistent with this result, *Aa*VA-1 but not *Aa*VA-9 specifically interacted with LRPPRC (Supplementary Fig. 13D). Overall, our results demonstrate that the interaction between *Aa*VA-1 and LRPPRC is specific and direct.

LRPPRC is a dominant negative interactor of Beclin-1 to suppress autophagy initiation^40–42^. We next investigated whether *Aa*VA-1 might release Beclin-1 from the LRPPRC-bound state. Indeed, LRPPRC co-precipitated with Beclin-1 using an anti-LRPPRC antibody. In the presence of purified recombinant *Aa*VA-1, the amount of Beclin-1 pulled down by LRPPRC reduced significantly; while a significant amount of *Aa*VA-1 was co-immunoprecipitated by LRPPRC (Supplementary Fig. 14A, left panel). We next precipitated Beclin-1 and its bound LRPPRC. The amount of LRPPRC bound by Beclin-1 was decreased by purified *Aa*VA-1 (Supplementary Fig. 14A, right panel). We also noted that Beclin-1 did not pull down *Aa*VA-1. The aforementioned study identified that *Aa*VA-1 directly interacts with the purified LRPPRC-T3 fragment (Supplementary Fig. 12G). We next asked if Beclin-1 also binds the T3 domain of LRPPRC using purified human Beclin-1. Indeed, Beclin-1 bound LRPPRC-T3, and this interaction was interrupted by *Aa*VA-1 (Supplementary Fig. 14B-C), further indicating that *Aa*VA-1 directly interacts with LRPPRC and displaces Beclin-1 by competing for the same binding motif in LRPPRC. We then examined whether native salivary *Aa*VA-1 had a similar function as the recombinant *Aa*VA-1. Indeed, Beclin-1 pulled down more LRPPRC in the presence of *Aa*VA-1-deficient SGE (Δ*Aa*VA-1-SGE) than WT-SGE (Fig. 5g). This phenomenon was further validated in the physiological condition. In human moMØ cells, native Beclin-1 colocalized with LRPPRC on mitochondria very well. The co-localizations were disrupted on mitochondria of the cells treated with WT-SGE, but remained intact in Δ*Aa*VA-1-SGE-treated cells (Fig. 5h). These results collectively indicate that *Aa*VA-1 competes with Beclin-1 for LRPPRC, thereby enabling to liberate Beclin-1 from the LRPPRC-bound state to initiate autophagy process (Supplementary Fig. 14D).

Indeed, Beclin-1 is not only involved in autophagy, but also LC3-associated phagocytosis (LAP)^46^. LAP can process engulfed particles, including pathogens, immune complexes, and dying cells, to regulate macrophage immune responses^17,46^. Unlike canonical autophagy, LAP does not require the components of the pre-initiation complex that trigger autophagy in response to nutrient stress (ULK1, FIP200, and ATG13). Nonetheless, LAP requires the Beclin-1 and VPS34 initiation complex that contains Rubicon (Run domain Beclin-1 interacting and cysteine-rich containing protein)^17,46^. We showed that Beclin-1 is released by *Aa*VA-1 from LRPPRC-mediated sequestration. We next investigated whether the released Beclin-1 could also take part in the LAP process, which may facilitate the viral entry. Indeed, siRNA-mediated knockdown of *FIP200*, a gene encoding an essential factor in canonical autophagy, reduced ZIKV infection in the *Aa*VA-1-incubated THP-1 cells (Supplementary Fig. 15A, B). However, silencing *Rubicon*, a participant in Beclin-1- mediated LAP, did not impair ZIKV load and even slightly enhanced the viral infection in the *Aa*VA-1 incubated cells (Supplementary Fig. 15C, D). We next assessed whether *Aa*VA-1-mediated release of Beclin-1 might regulate viral entry or assembly of the flavivirus replication compartment. First, we assessed whether Beclin-1 influenced ZIKV attachment on the surface of human immune cells. At 4 °C the virions are tethered on the cell surface, but not internalized^47^. Compared to the mock control, genetic suppression of *Beclin-1* did not influence ZIKV attachment on the surface of THP-1 cells (Supplementary Fig. 15E). We next investigated the role of Beclin-1 in viral internalization. ZIKV was incubated with either the *Beclin-1*-silenced THP-1 cells or the control siRNA-treated cells at 37 °C for 2 h, and viral loads were quantified after stringent washes. The viral load was not affected by knockdown of *Beclin-1* (Supplementary Fig. 15F). To further address that, we assessed the effect of both viral attachment and internalization in THP-1 cells with or without *Aa*VA-1 incubation. *Aa*VA-1 did not regulate ZIKV attachment and internalization (Supplementary Fig. 15G, H), indicating that *Aa*VA-1 does not play a role in autophagy-related phagocytosis. In addition, previous studies have indicated that HCV utilizes autophagosomal membranes as sites for its RNA replication^33^. However, ZIKV dsRNA, which represents the viral replication compartment, did not co-localize with the LC3B-positive puncta in the *Aa*VA-1-incubated macrophages, suggesting that the *Aa*VA-1-mediated initiation of autophagy may not directly affect the assembly of the flaviviral replication compartment (Supplementary Fig. 16A). Overall, we concluded that *Aa*VA-1-mediated Beclin-1 release facilitates flaviviral infection by activating canonical autophagy but not by initiating LAP-mediated viral entry or the assembly of the flavivirus replication compartment.

**Fig. 6:**
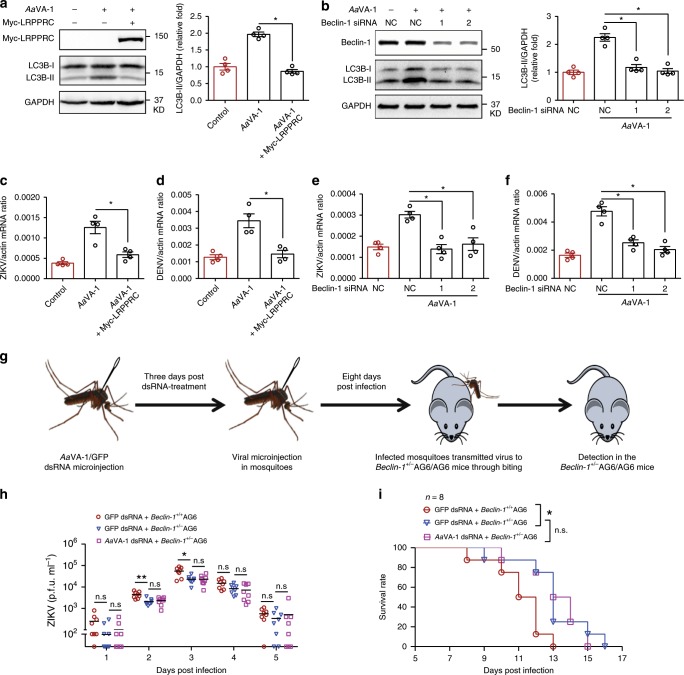
*Aa*VA-1-mediated Beclin-1 signaling activation facilitates viral transmission. **a**, **b** Ectopic expression of LRPPRC or siRNA-mediated knockdown of *Beclin-1* impaired the *Aa*VA-1-mediated autophagy activation in human THP-1 cells. **a** Purified *Aa*VA-1 (1 μg/ml) was incubated with either LRPPRC-expressing or mock cells for 6 h. **b** Purified *Aa*VA-1 (1 μg/ml) was incubated with either *Beclin-1*-silenced or mock cells for 6 h. Autophagy activity was detected by western blotting with anti-LC3B antibody (left panel), and quantification of the signaling intensity of LC3B-II relative to GAPDH (right panel). *n* = 4 independent experiments. **c**–**f** Either ectopic expression of LRPPRC (**c**, **d**) or siRNA-mediated knockdown of *Beclin-1* (**e**, **f**) restored the enhancement of DENV and ZIKV infectivity caused by *Aa*VA-1. Purified *Aa*VA-1 (1 μg/ml) was incubated with the cells. The same amount of BSA served as a negative control. The MOI for infection was 0.1 for DENV and ZIKV. Gene quantities were normalized against human *actin* (NM_001101.4). *n* = 4 independent samples. The experiments were repeated 3 times with the similar results. **g**–**i** A deficiency of Beclin-1 in the host offsets *Aa*VA-1-mediated ZIKV transmission by *A. aegypti*. **g** Schematic representation of the study design. The *AaVA-1* gene was silenced by dsRNA thoracic microinjection in *A. aegypti*. Mosquitoes inoculated with *GFP* dsRNA served as mocks. Three days post gene silencing, the dsRNA-treated mosquitoes were thoracically infected with ZIKV. After an additional 8 days of rearing, either *Beclin-1*^*+/−*^AG6 or the *Beclin-1*^*+/+*^AG6 mouse were subjected to a blood meal by the infected mosquitoes. Both ZIKV viremia and mortality were recorded daily after mosquito biting. **h** The ZIKV load in mouse plasma was detected by a plaque assay. **i** Animal mortality was recorded daily (*n* = 8 mice per group). The data were combined based on two independent experiments. **a**–**f, h** The data are presented as the mean ± SEM. A nonparametric Mann–Whitney test was used for the statistical analysis. *p < 0.05, **p < 0.01, n.s., not significant (*p* ≥ 0.05). **i** Survival rates of the infected mice were statistically analysed using the log-rank (Mantel–Cox) test. Source data are provided as a Source Data file.

We next assessed whether genetic manipulation of LRPPRC and Beclin-1 influenced *Aa*VA-1-mediated autophagy activation. In human cells, *Aa*VA-1 treatment increased LC3B-II levels and viral (ZIKV/DENV) replication, which were however inhibited by either ectopic expression of LRPPRC (Fig. 6a, c, d) or siRNA-mediated knockdown of *Beclin-1* (Fig. 6b, e, f). Next, we assessed these phenotypes using a gene knockout mouse model. Given the essential roles of these autophagy factors in animal development^48^, neither *lrpprc*^*–/–*^ nor *Beclin-1*^*–/–*^ mice survive the embryonic stage^45,49^. Nonetheless, heterozygous *Beclin-1*^*+/–*^ mice develop normally. We therefore generated a *Beclin-1*^*+/–*^*ifnar1*^*–/–*^*ifngr1*^*–/–*^ (*Beclin-1*^*+/–*^AG6) mouse line by cross-breeding the *Beclin-1*^*+/–*^ and *ifnar1*^*–/–*^*ifngr1*^*–/–*^ strains (Supplementary Fig. 17A, B). The *Beclin-1*^*+/–*^AG6 mice showed a lower autophagy activity than the *Beclin-1*^*+/+*^AG6 control mice (Supplementary Fig. 17C). We next assessed whether *Aa*VA-1 promoted ZIKV transmission to the Beclin-1-deficient mice, through an “*A. aegypti*-mouse” transmission model. The *AaVA-1* gene was silenced by thoracic microinjection of dsRNA in *A. aegypti*, and subsequently thoracically infected with ZIKV. After rearing for an additional 8 days, both the *Beclin-1*^*+/–*^ and *Beclin-1*^*+/+*^ AG6 mice were subjected to a blood meal by the infected mosquitoes (Fig. 6g). Both ZIKV viremia and mortality were recorded daily after mosquito biting. The *Beclin-1*^*+/–*^AG6 mice (blue) had lower ZIKV viremia (Fig. 6h) and prolonged survival (Fig. 6i) than the *Beclin-1*^*+/+*^AG6 mice (red) bitten by *GFP*-dsRNA-treated ZIKV-carrying mosquitoes. However, the *Beclin-1*^*+/–*^AG6 mice that were bitten by the *AaVA-1* dsRNA-treated mosquitoes (violet) did not present any significant difference in ZIKV viremia or mortality compared with the *GFP* dsRNA-treated mosquitoes (blue) (Fig. 6h, i). These data indicate that the pro-viral effect of *Aa*VA-1 in flaviviral transmission is dependent on the Beclin-1-mediated autophagy.

## Discussion

Mosquito-borne flaviviruses have evolved sophisticated strategies to efficiently survive in two dramatically different environments^7,29^. Viral transmission from an infected mosquito to a susceptible mammalian host is an essential step in the flaviviral lifecycle. Accumulated evidence indicates that mosquito salivary proteins can manipulate host cellular and immune responses, which enhances viral transmission efficiency^15,16,30,50^. A 34 kDa protein from *A. aegypti* saliva enhances DENV replication in human keratinocytes by suppressing interferon expression^28^. Recently, a study has shown that an *A. aegypti* salivary factor LTRIN could facilitate ZIKV transmission by interfering with nuclear factor-κB signaling and the downstream inflammatory cytokine production^30^. An antiserum against *A. aegypti* salivary protein AgBR1 partially protected mice from lethal mosquito-borne ZIKV infection by suppressing early inflammatory responses in skin^50^. Passive immunization against *A. aegypti* NeSt1 prevented macrophages from infiltrating the bite site and ameliorated ZIKV-induced pathogenesis in mice^31^. In addition to regulating host immune responses, mosquito saliva may enhance viral infection of host cells directly. A serine protease in *A. aegypti* saliva has been shown to augment DENV infection by proteolyzing extracellular matrix proteins, thereby increasing viral attachment to heparan sulfate proteoglycans and inducing cell migration^16^. In this study, we characterized the *A. aegypti* salivary proteins by mass spectrometry and identified *Aa*VA-1, a female *Aedes* mosquito saliva-specific protein, as a pro-viral factor for DENV and ZIKV transmission. *Aa*VA-1 promotes flaviviral replication in human monocyte-lineage immune cells, the primary intradermal target of flaviviruses during mosquito biting^8,9,11^. Inoculation of *Aa*VA-1 with the viruses leads to robust viremia and disease onset, whereas *Aa*VA-1 depletion in infected *Aedes* mosquitoes results in a lower viral load and prolonged lifespan in the bitten animals. Mechanistic studies indicate that *Aa*VA-1 can gain access into a human immune cell in a RhoA-dependent manner, intracellularly trafficking from endosomes to mitochondria. *Aa*VA-1 sequesters LRPPRC on mitochondria, an autophagy antagonist, thereby activating autophagy to promote DENV and ZIKV replication in human immune cells. Indeed, LRPPRC has been identified as a Beclin-1 suppressor to prevent formation of the Beclin-1-PI3KCIII autophagy initiator^40–42^. *Aa*VA-1 offsets LRPPRC-mediated inhibition of Beclin-1, thereby enabling the downstream autophagy activation.

Previous studies have shown that LRPPRC posttranscriptionally regulates mitochondrial mRNAs inside mitochondria^44,51^. Nonetheless, abundant evidence indicates that LRPPRC can interact with cytosolic proteins. For example, HCV NS5A localizes to the endoplasmic reticulum during viral infection, while the NS5A binds to LRPPRC on mitochondria to inhibit the MAVS-regulated antiviral signaling during HCV infection^52^. LRPPRC plays a direct role in eIF4E-dependent mRNA export by directly interacting with eIF4E, which localizes to both nucleus and cytoplasma^53^. Intriguingly, the LRPPRC/SLIRP complex has a specific role in mRNA maturation after transcription in mitochondria. LRPPRC may shuttle between a soluble matrix fraction and an inner membrane fraction that might depend on its association with SLIRP^54^. We therefore propose that LRPPRC not only localizes inside mitochondria, but also may be present on the surface of mitochondria to interact with cytoplasmic proteins for their functions.

*Aa*VA-1 gains access into a human immune cell by a RhoA-dependent endocytosis and then intracellularly traffics from endosomes to mitochondria. Indeed, some external toxin proteins can be acquired intracellularly by an endocytic route, and then escape from endosome to localize in organelles of cytosol. For example, the purified Tat protein from human immunodeficiency virus 1 can be taken up by cells and subsequently transactivate the viral promoter^55,56^. An antennapedia homeobox peptide, pAntp, can penetrate into the cells and regulate neural morphogenesis^57^. Shiga toxin B-fragment can enter cells through both CME and clathrin-independent endocytosis. This protein is directly transported to the Golgi network from early endosome^58^, and then reach the ER, finally translocating in the cytosol^59^. Ricin A-chain, another external toxin protein, is also transported to the cytosol^60^. Thus, we speculate that *Aa*VA-1 might utilize the similar aforementioned strategy.

Autophagy is a cellular process that responds to stress stimuli to maintain cellular homeostasis^48^. Besides, autophagy plays either pro- or antiviral roles during viral infection. Previous studies indicated the important roles of autophagy in host defense against pathogens and positive regulation of immune responses^61^. For example, activation of autophagy facilitates innate recognition of viral pathogens and IFN-α production in vesicular stomatitis virus-infected plasmacytoid DCs. The viral recognition by TLR7 requires transportation of cytosolic viral intermediates into lysosome by the process of autophagy^62^. Autophagy is essential for TLR3-mediated type I interferon production in the Coxsackie virus B3-infected kidney fibroblasts^63^. Nonetheless, some pathogens have evolved mechanisms to usurp autophagy to facilitate their robust replication in the host. The replication of several viruses in the *Picornaviridae* family is suppressed by the presence of autophagy inhibitors^64,65^. In addition, infections by many members of the *Flaviviridae* family, such as HCV^33^, DENV^22^, JEV^32^ and ZIKV^24^, are able to activate autophagy to promote the replication of these viruses and the maturation of viral particles.

In our experimental settings, manipulation of *Aa*VA-1 resulted in a small change in viral loads; nonetheless, the *Aa*VA-1-treated animals succumbed to infection 2–3 days earlier than mock-treated animals. Therefore, *Aa*VA-1 might promote viral pathogenesis in animals beyond enhancement of viral replication. Mosquito saliva is able to regulate the host inflammatory immune responses^28,30,50^. We found that incubation of *Aa*VA-1 in THP-1 cells did not influence the expression of TNF-α, IL-1β, IFN-α2, IFN-α7, IL-8, MCP-1, IFN-β, or MIP-1α. However, we cannot exclude the possibility that *Aa*VA-1 plays a regulatory role in other immune responses. In this study, we showed that *Aa*VA-1 activates autophagic signaling in human immune cells. Indeed, autophagy is incorporated into adaptive immune pathways through the regulation of antigen presentation and lymphocyte homeostasis^66^. Intriguingly, previous studies found that mosquito bites promoted a Th2 cell-dominated immune response to enhance virus replication^67^, suggesting that *Aa*VA-1 might play multiple roles in facilitating flavivirus pathogenesis in mammals in addition to enhancing viral replication.

Intriguingly, mosquito-borne flaviviruses are specifically carried and transmitted by different mosquito species. The *Aedes* mosquitoes show higher vector competence for DENV and ZIKV transmission, while some other flaviviruses such as JEV and WNV are predominantly transmitted by the *Culex* mosquitoes in nature^68^. The salivary components of the two mosquitoes also differ substantially. For example, a previous study has indicated that the SGE from the mosquitoes of *A. aegypti* and *Culex quinquefasciatus* differentially modulate host immune responses^69^. *C. quinquefasciatus* saliva exerts an inhibitory effect on platelet-activating factor (PAF) and has salivary PAF-specific phospholipase C activity, whereas *A. aegypti* SGE does not show any similar activities^70^. We found that *Aa*VA-1 acts as positive regulators of autophagy, thereby promoting DENV and ZIKV replication in host cells. Indeed, a homologue of *Aa*VA-1 was identified from *C. quinquefasciatus* by sequence comparison (43% amino acid identity to *Aa*VA-1). Contribution of *Culex* VA-1 in JEV and WNV transmission by inducing autophagy remains to be further understood.

Mosquito saliva may promote viral transmission in multiple ways, such as modulating host inflammatory responses, suppressing IFN signaling and promoting viral attachment. In this study, we discovered a role of mosquito saliva-mediated autophagy activation in facilitating flaviviral transmission. This study may provide insights into the molecular basis of flaviviral survival in their lifecycle and the results could offer therapeutic targets for the prevention of flavivirus dissemination in nature.

## Methods

### Ethics statement

Human blood was collected from healthy donors who provided written informed consent. The collection of human blood samples was approved by the local ethics committee at Tsinghua University.

### Mice, mosquitoes, cells, and viruses

AG6 mice were donated by the Institute Pasteur of Shanghai, Chinese Academy of Sciences. *Beclin-1*^*+/–*^ mice (Cat# 018429) were purchased from the Jackson Laboratory. The mice were bred and maintained under a specific pathogen-free animal facility at Tsinghua University. Groups of age- and sex-matched mice, 8–10 weeks of age, were used for the animal studies. All animal protocols used in this study were approved by the Institutional Animal Care and Use Committee of Tsinghua University and performed in accordance with their guidelines. The laboratory animal facility has been accredited by AAALAC (Association for Assessment and Accreditation of Laboratory Animal Care International). *A. aegypti* (the Rockefeller strain) and *A. albopictus* (the Jiangsu strain) were reared in a low-temperature, illuminated incubator (Model 818, Thermo Electron Corporation) at 28 °C and 80% humidity, according to standard rearing procedures^29^. ZIKV (GZ01 strain, KU820898) and DENV-2 (New Guinea C strain, AF038403.1) were used in the experiments. The viruses were passaged in *A. albopictus* C6/36 cells. The virus titers were determined by a plaque formation assay. The *Drosophila melanogaster* S2 cell line was cultured in Schneider’s medium with 10% heat-inactivated fetal bovine serum (Cat# 16000-044, Gibco) and 1% antibiotic-antimycotic (Cat# 15240-062, Invitrogen). The Vero cells and 293T cells were maintained in Dulbecco’s modified Eagle’s medium (Cat# 11965-092, Gibco) supplemented with 10% heat-inactivated fetal bovine serum. The THP-1 cells were maintained in Roswell Park Memorial Institute (RPMI) 1640 medium (Cat# 22400089, Gibco) supplemented with 10% heat-inactivated fetal bovine serum. The Vero, 293T, C6/36 and THP-1 cell lines were purchased from the ATCC (Cat# CCL81, CRL-3216, CRL-1660 and TIB-202). The *Drosophila* S2 cell line (Cat# R690-07) and FreeStyle-293F Cells (Cat# R790-07) were provided by Invitrogen.

### Western blotting analysis and antibodies

The *AaVA-1* gene was amplified from the complementary DNA of mosquitos and cloned into a pET-28a (+) expression vector (Cat# 69864, Millipore). The cloning primers are presented in Supplementary Table 4. Recombinant *Aa*VA-1 protein was expressed in the *Escherichia coli* BL21 DE3 strain in an insoluble form in inclusion bodies. The protein was then solubilized in 8M urea and purified with a TALON Purification Kit (Cat# 635515, Clontech). Mouse antiserum was produced via inoculation with recombinant *Aa*VA-1 with three boosts, and the serum was divided and stored at −80 °C until further use. The samples were separated by 12% SDS–PAGE gel, followed by electrophoretic transfer to polyvinylidene fluoride membranes, and blocking and incubating membranes with primary antibodies. The following antibodies were used in these experiment: anti-LC3B (Cat# 2775, 1:1000 dilution), anti-FIP200 (Cat# 12436, 1:1000 dilution), and anti-GAPDH (Cat# 2118, 1:3000 dilution) antibodies were obtained from Cell Signaling Technology (CST). Anti-Beclin-1 (Cat# 66665-1-Ig, 1:2000 dilution), anti-RhoA (Cat# 10749-1-AP, 1:1000 dilution), anti-Rubicon (Cat# 21444-1-AP, 1:1000 dilution), anti-Actin (Cat# 60008-1-Ig, 1:2000 dilution), and anti-LRPPRC (Cat# 21175-1-AP, 1:1000 dilution) antibodies were obtained from the Proteintech Group. Anti-V5 (Cat# M167-3, 1:1000 dilution) and anti-Myc (Cat# 562, 1:1000 dilution) antibodies were purchased from Medical & Biological Lab (MBL). Anti-*Aa*VA-1 (mouse serum) was used at 1:1000 dilution. Anti-V5-HRP (Cat# R961-25, dilution 1:5000) and anti-Myc-HRP (Cat# R951-25, 1:5000 dilution) antibodies were purchased from Invitrogen. Uncropped scans of blots have been provided in the Supplementary Information.

### Expression and purification of recombinant protein

The genes encoding *A. aegypti* salivary protein were cloned without signal sequences into a pMT/BiP/V5-His A vector (Cat# V4130-20, Invitrogen) for expression in *Drosophila* S2 cells. For immunoprecipitation assays, the human *LRPPRC* gene, *LRPPRC* truncations, *PTCD1* gene, and *Beclin-1* gene were cloned into a pcDNA3.1/Myc-His C vector for expression in 293T cells. LRPPRC and LRPPRC-T3 were purified from 293F cells using a Cobalt-His column. Beclin-1 was cloned into a pGEX-6P-2 vector and purified from *E. coli* using glutathione sepharose. The cloning primers are shown in Supplementary Table 4. For *Aa*VA-1 expression, the stable S2 cells were cultured in S2 Schneider’s medium in a 175 cm^2^ flask and then transferred into spinner flasks containing Express Five serum-free medium (Cat# 10486-025, Gibco) for protein expression. The cells were further cultured for 3 days and induced with 500 μM copper sulfate for another 4 days. The supernatant was centrifuged, filtrated, and then concentrated for *Aa*VA-1 purification by a TALON Purification Kit (Cat# 635515, Clontech).

### Gene silencing and viral infection in mosquitoes

For silencing the target genes, female *Aedes* mosquitoes were maintained on ice for 15 min, and then transferred to a cold tray (Cat# 1431, BioQuip) to receive a thoracic injection of dsRNA into the hemocoele. In addition, 1 μg/300 nl of dsRNA was microinjected into mosquito thoraxes. Subsequently, the injected mosquitoes were allowed to recover under standard rearing conditions for further investigation. *A. aegypti* salivary glands were dissected and the SGE was generated from the gene-silenced *A. aegypti*. The gene silencing efficiency was assessed by quantitative reverse transcriptase PCR (qRT-PCR) and western blotting analysis. The primers used for gene detection are shown in Supplementary Table 4. For viral infection in mosquitoes, 1000 M.I.D._50_ (50% mosquito infectious dose) of ZIKV was microinjected into the mosquito thoraxes. The infected mosquitoes were subjected to biting the AG6 mice for further investigation.

### qPCR detection

Total RNA was isolated from homogenized mosquitoes using an RNeasy Mini Kit (Cat# 74106, Qiagen) and reverse-transcribed into cDNA using an iScript cDNA synthesis kit (Cat# 170-8890, Bio-Rad). qRT-PCR was performed on the Bio-Rad CFX-96 Touch Real-Time Detection System. Primer sequences are shown in Supplementary Table 4.

### Determination of the virus in infected mice

Blood samples were collected from the tail veins of infected mice in 0.4% sodium citrate and centrifuged for 5 min at 6,000 × *g* and 4 °C to isolate plasma. The presence of infectious viral particles in the plasma was determined using a plaque assay^29^.

### Enzyme-linked immunosorbent assay

The purified proteins were used in an enzyme-linked immunosorbent assay (ELISA) assay. The prey protein (2 μg) was coated overnight at 4 °C. The plates were blocked using 5% w/v bovine serum albumin (BSA) solution for 1 h at room temperature. The bait proteins were added and incubated for 2 h. After washing, the primary antibody was added and incubated for an additional 2 h. After washing again, a secondary IgG-HRP was added to each well, and the plates were incubated for 1 h. The commercial peroxidase substrate system was used for signaling detection (Cat# 5120-0053, SeraCare), and the optical density at 450 nm was measured with an ELISA reader.

### Co-immunoprecipitation

The recombinant plasmids were transiently transfected into 293T cells with a Lipofectamine 2000 transfection reagent (Cat# 11668019, Invitrogen). After 48 h of transfection, co-IP was performed by a Pierce Classic IP Kit (Cat# 26146, Thermo Scientific). The cells were incubated on ice with the IP lysis buffer containing a protease inhibitor cocktail (Cat# 4693132001, Roche). The cell lysates was incubated with 1 μg of purified *Aa*VA-1 at 4 °C, subsequently the mixture was incubated with 2 μg of antibody overnight. The Protein A/G Agarose was added for 2 h incubation. After washing with the IP lysis buffer, the samples were detected by western blotting.

### Immunofluorescence staining and microscopy

For the *Aa*VA-1 entry and binding assays, purified *Aa*VA-1 (5 μg/ml) was incubated with human immune cells. The cells were washed three times in phosphate-buffered saline (PBS) and fixed with ice-cold methanol (−20 °C) or 4% paraformaldehyde for 15 min following the antibody instructions. The cells were then permeabilized with 0.3% Triton X-100 for 20 min, washed with PBS, and blocked in 2% BSA for 1 h. The cells were stained with anti-V5 (Cat# M167-3, MBL), anti-LRPPRC (Cat# 21175-1-AP, Proteintech), anti-Beclin-1 (Cat# 66665-1-Ig, Proteintech), anti-Cdc42 (Cat# ab64533, Abcam), anti-RhoA (Cat# 10749-1-AP, Proteintech), anti-Clathrin (Cat# 4796, CST), anti-Caveolin-1 (Cat# 3267, CST), anti-Flotillin-1 (Cat# 18634, CST), anti-Arf6 (Cat# PA1-093, Invitrogen), anti-Rab5 (Cat# 2143, CST), anti-Rab7 (Cat# 9367, CST), anti-Lamp1 (Cat# 9091, CST), anti-GM130 (Cat# 12480, CST), anti-Tom20 (Cat# 42406, CST), anti-Calnexin (Cat# 2679, CST), anti-dsRNA J2 (Cat# 10010200, SCICONS), anti-*Aa*VA-1 (mouse serum), anti-flavivirus E protein (4G2), and anti-LC3B (Cat# 2775, CST) as 1:200 dilution at 4 °C overnight, according to different experimental settings. The cells were washed and then stained with fluorescence-conjugated secondary antibodies for 1 h at room temperature. The nuclei were stained blue with DAPI (Cat# 10236276001, Roche). For staining LRPPRC, *Aa*VA-1 and Beclin-1 on mitochondria, biotin-labeled *Aa*VA-1 (5 μg/ml), anti-LRPPRC (Cat# sc166178, Santa Cruz, 1:200 dilution), or anti-Beclin-1 (Cat# AF5295, R&D Systems, 1:200 dilution) were used for the experiments. Images were examined using a Zeiss LSM 780 meta confocal microscope.

### siRNA transfection

Both siRNA oligos against *Beclin-1*, *RhoA, FIP200*, and *Rubicon* genes and the negative siRNA were ordered from Shanghai GenePharma. siRNA sequences are listed in Supplementary Table 4. siRNA oligos were transfected into THP-1 cells using Cell Line Nucleofector Kit V (Cat# VCA-1003, Lonza) following the manufacturer’s instructions.

### Human moDC and moMØ generation

Peripheral blood mononuclear cells (PBMCs) were isolated by Ficoll-Paque density gradient centrifugation (Cat# 17144002, GE Healthcare) from healthy donor blood. Human moDC and moMØ were generated from PBMCs by adherence to plastic for 2 h at 37 °C in 5% CO_2_. For moDC generation, monocytes were cultured in RPMI 1640 medium supplemented with 10% heat-inactivated fetal bovine serum, 100 ng/ml granulocyte–macrophage colony-stimulating factor (GM-CSF) (Cat# 34-8339-82, eBioscience) and 100 ng/ml IL-4 (Cat# 34-8049-82, eBioscience) for 7 days. For macrophage generation, monocytes were cultured in RPMI 1640 medium supplemented with 10% heat-inactivated fetal bovine serum and 100 ng/ml GM-CSF for 7 days.

### Quantification and statistical analysis

Animals were randomly allocated into different groups. Mosquitoes that died before measurement were excluded from the analysis. The investigators were not blinded to the allocation during the experiments or to the outcome assessment. Descriptive statistics have been provided in the figure legends. Given the nature of the experiments and the type of samples, differences in continuous variables were assessed with the nonparametric Mann–Whitney test. The survival rates of the infected mice were statistically analysed using the log-rank (Mantel–Cox) test. All analyses were performed using the GraphPad Prism statistical software.

### Reporting summary

Further information on research design is available in the Nature Research Reporting Summary linked to this article.

## Supplementary information

Supplementary Information

Reporting Summary

## Data Availability

All data generated or analyzed during this study are included in this published article and its Supplementary Information files. The source data underlying Figs. 1c–f, 2b–g, 3b, c, e, g–i, 4a–f, 5b, c, f, h, 6a–f, h, i, and Supplementary Figs. 1B-E, 2, 3, 5, 6C-E, 6G-I, 7, 8, 9, 10, 12G, 13B, 14C,15B, D and E-H are provided as a Source Data file. Uncropped scans of blots/gels of Figs. 1a, b, 2a, 3f, 4a, 5e, g, 6a, b, and Supplementary Fig. 4, 6B, 6F, 11B, 12A, C, E, F, 13C, D, 14A, B, 15A, C, and 17B-C have been provided in Supplementary Figs. 18–20.

## Source data

Source Data
