# Supplementary material for: A mosquito salivary protein promotes flavivirus transmission by activation of autophagy
Source: Nat Commun. 2020 Jan 14;11:260. doi: 10.1038/s41467-019-14115-z (PMC6959235; doi:10.1038/s41467-019-14115-z)
Supplement: Supplementary file 1 — Supplementary Information [file 41467_2019_14115_MOESM1_ESM.pdf]

Supplementary Information

A mosquito salivary protein promotes flavivirus transmission by activation of autophagy

Sun et al

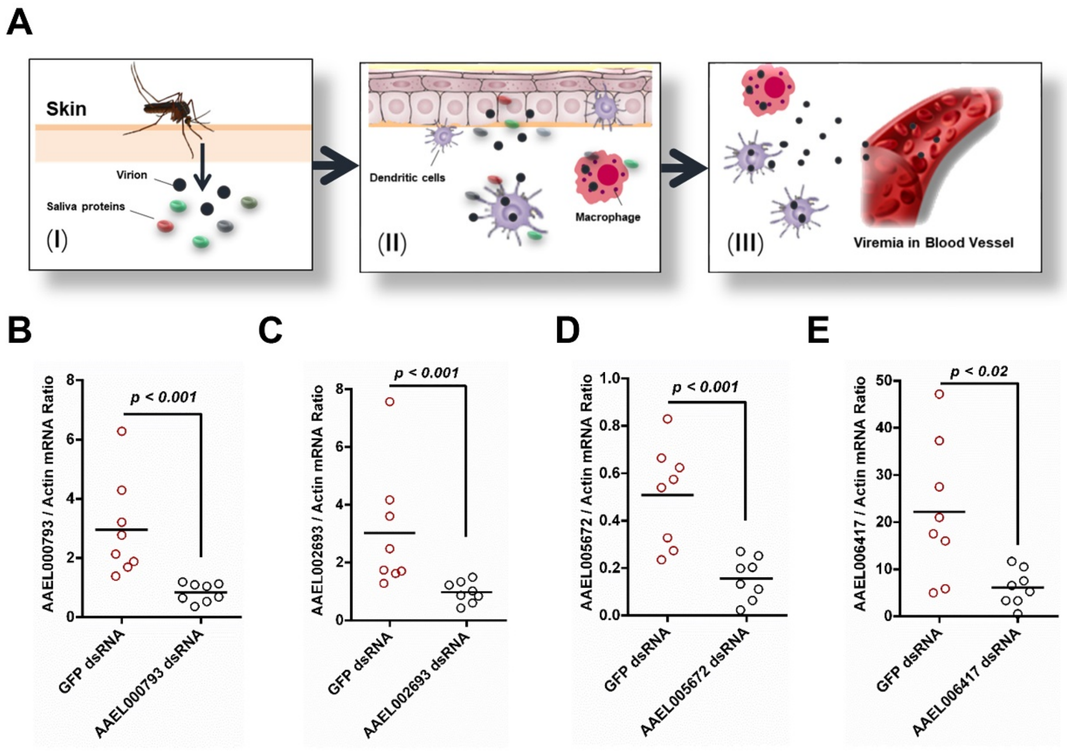

Supplementary Figure 1. Schematic representation of flavivirus transmission, and knockdown efficiency of genes in the mosquito salivary glands. (A) Schematic representation of flavivirus transmission by mosquito biting. (i) The viruses were inoculated with salivary proteins by mosquito biting. (ii) The viruses inoculated by mosquitoes primarily infect dermal-resident monocyte-lineage cells, such as immature DC subsets and macrophages. Mosquito salivary proteins, inoculated together with the infectious particles, may directly interact with host cells to regulate the initial viral replication. (iii) the viruses are released from the primary infected cells into the blood circulation, thereby causing subsequent viremia and disease. (B-E) Silencing genes in the mosquito salivary glands. At 6 days after dsRNA treatment, the mosquitoes were sacrificed to determine silencing effect by qRT-PCR and normalized by *A. aegypti actin* (*AAEL01119*). The primers of dsRNA synthesis and qRT-PCR detection were described in Supplementary Table 4. One dot represented 1 mosquito and the horizontal line represented the mean value in all figures. We used the Mann-Whitney test for statistical analysis. $n = 8$ independent samples. The experiment was repeated three times with the similar results. Source data are provided as a Source Data file.

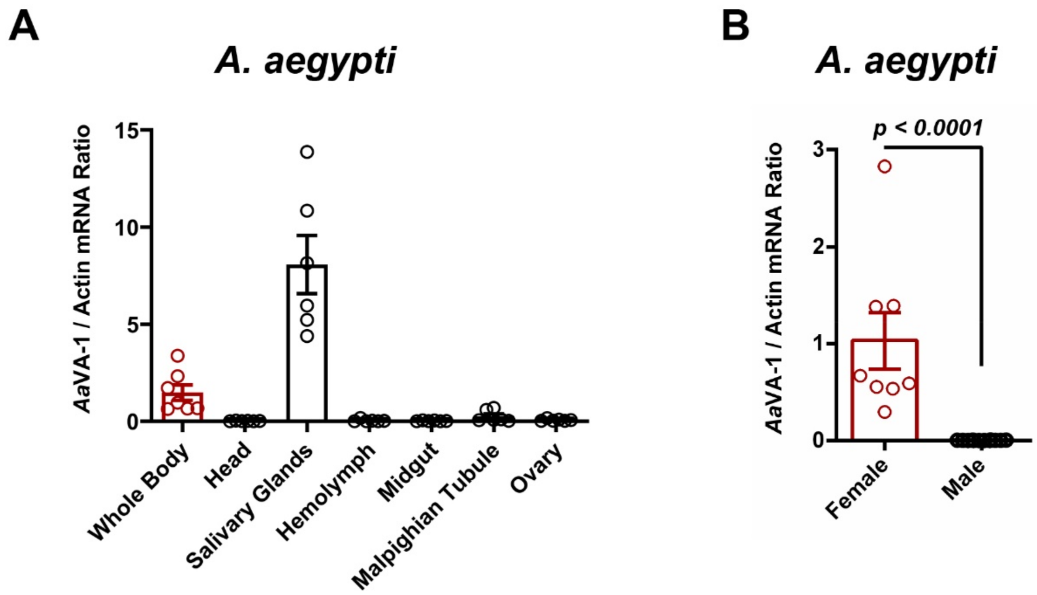

Supplementary Figure 2. ***Aa*****VA-1 is a female mosquito salivary gland-specific protein**. (A) Expression of *AaVA-1* in various tissues of *A. aegypti*. (B) Expression of *AaVA-1* in female or male *A. aegypti* mosquitoes. (A-B) Gene quantities were normalized against *A. aegypti actin* (*AAEL01119*). The data are presented as the mean ± s.e.m. A nonparametric Mann-Whitney test was used for the statistical analysis. (A) n = 6-7 independent samples. (B) n = 8 (Female) or n = 12 (male) independent samples. The data were combined based on 2 independent experiments. Source data are provided as a Source Data file.

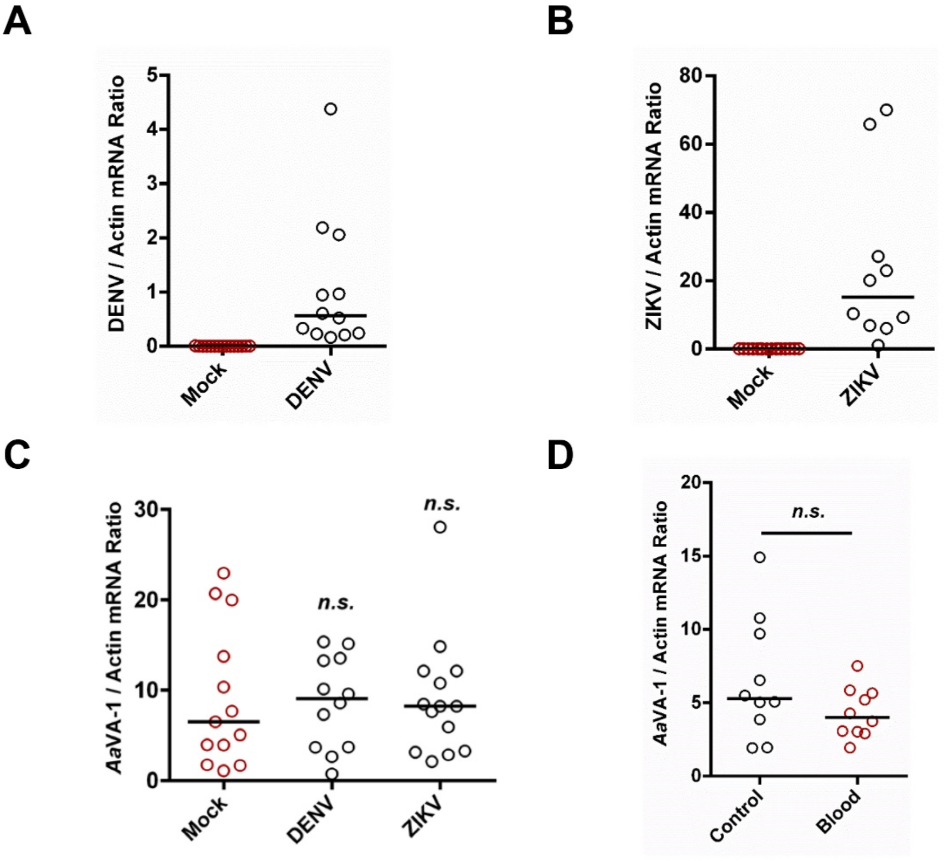

Supplementary Figure 3. *Aa*VA-1 cannot be induced by flaviviral infection and blood meal. DENV or ZIKV was thoracically inoculated into female *A. aegypti* mosquitoes. The salivary glands were dissected at 8 days post-infection. Both DENV (A) and ZIKV (B) infectivity and *Aa*VA-1 expression in the salivary glands of *A. aegypti* were detected by qRT-PCR (C). (D) *Aa*VA-1 cannot be induced by a blood meal. The salivary glands were dissected at 4 days post blood meal for qRT-PCR detection. Gene quantities were normalized against *A. aegypti actin* (*AAEL011197*). (A-D) Each dot represents a pair of mosquito salivary glands, and each line represents the median value of the group. A nonparametric Mann-Whitney test was used for the statistical analysis. *n.s.*, not significant ($p \geq 0.05$). (A) n = 15 (Mock) or n = 12 (DENV) independent samples. (B) n = 16 (Mock) or n = 10 (ZIKV) independent samples. (C) n = 13 (Mock), n = 12 (DENV) or n = 14 (ZIKV) independent samples. (D) n = 10 independent samples. The experiment was repeated 3 times with the similar results. Source data are provided as a Source Data file.

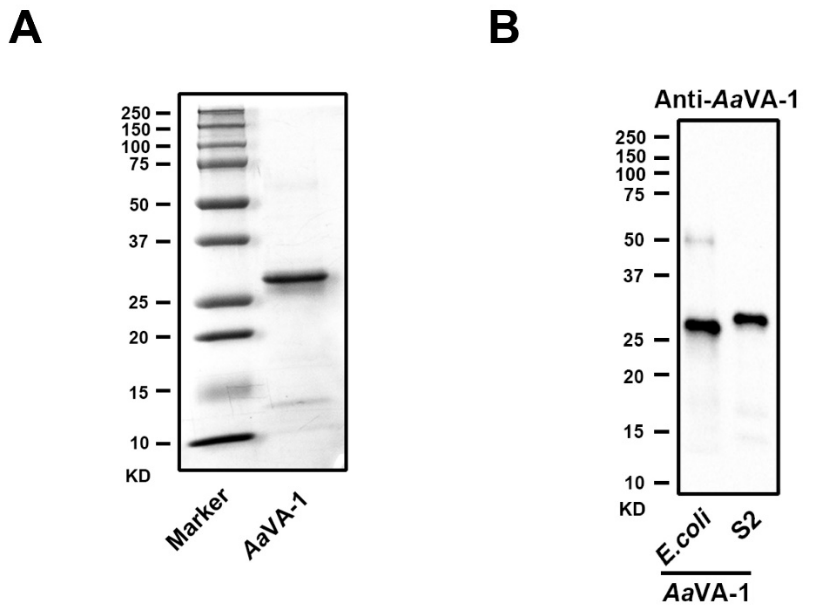

Supplementary Figure 4. Generation of a murine *Aa*VA-1 polyclonal antibody. (A) Purification of *Aa*VA-1 recombinant protein in *E. coli*. *AaVA-1* was cloned into the pET-28a (+) DNA vector and expressed in the *E. coli* BL21 DE3 strain. The recombinant protein, expressed in inclusion bodies, was dissolved in 8 M urea and purified for antibody generation. (B) Validation of the *Aa*VA-1 polyclonal antibody. A murine *Aa*VA-1 polyclonal antibody was used to probe *E. coli*- or S2-expressed *Aa*VA-1 recombinant proteins. The experiments were repeated 3 times with the similar results.

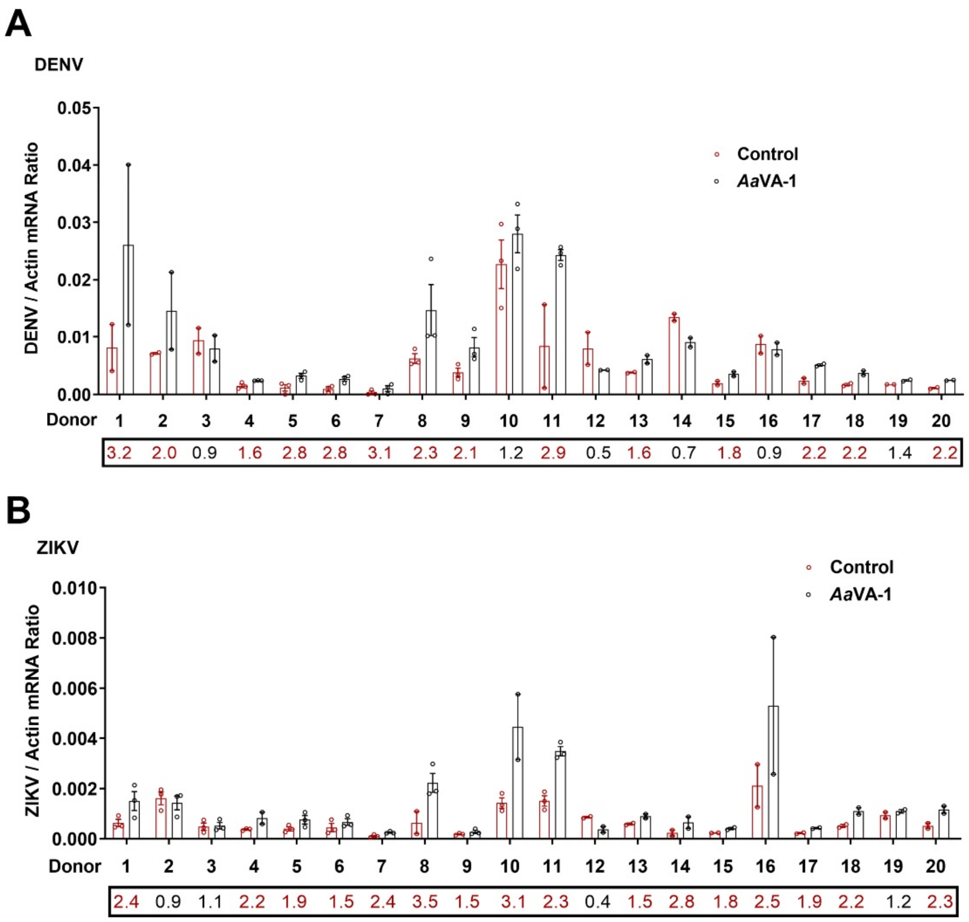

Supplementary Figure 5. Assessment of *Aa*VA-1-mediated flaviviral infection in human monocytes. The human monocytes were isolated from 20 human individual donors. *Aa*VA-1 (1 μg/ml) was mixed with DENV (0.1 MOI) or ZIKV (0.1 MOI) to infect monocytes from each donor. An equal amount of BSA with viruses served as mock controls. Viral infectivity was assessed by qRT-PCR at 24 h post-infection. Gene quantities were normalized against human *actin* (*NM_001101.4*). The number in the box below the donor numbers represents the fold infectivity of the infected cells with vs without *Aa*VA-1. The data are presented as the mean ± s.e.m. n = 2-3 independent samples. Source data are provided as a Source Data file.

A

Species	Gene Number	Length (AA)	Identity
Aedes albopictus	AALF011740	256	78%
Aedes aegypti	AAEL000793	255	/

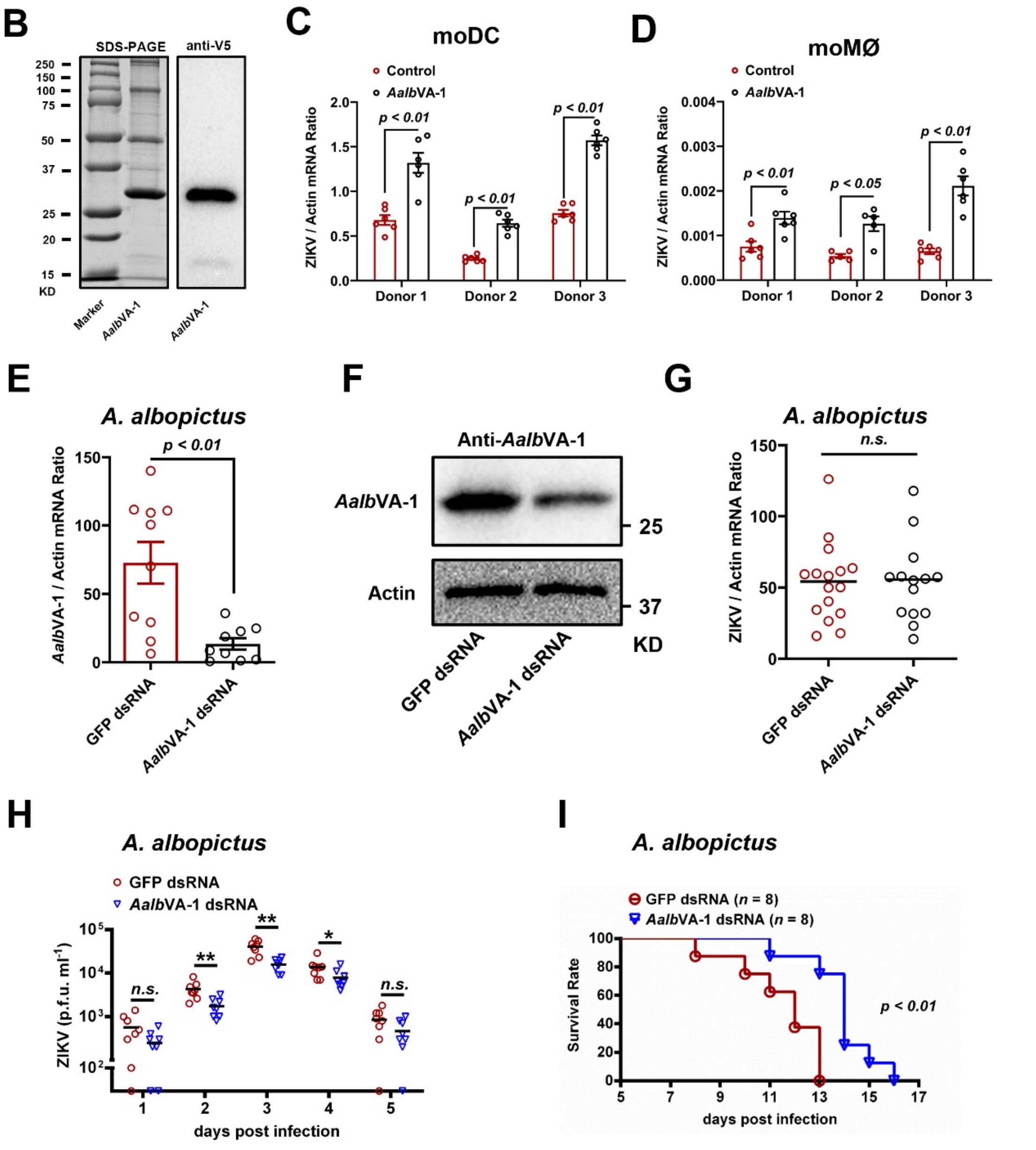

Supplementary Figure 6. *A. albopictus* VA-1 promotes ZIKV transmission (A) Identification of the homologue of *Aa*VA-1 in *A. albopictus*. (B) Expression and purification of *Aalb*VA-1 using a *Drosophila* expression system. The recombinant *Aalb*VA-1 was expressed and purified using a Cobalt-His column (left panel). The expression was probed with anti-V5 antibody (right panel). (C-D) Incubation of *Aalb*VA-1 enhanced the replication of ZIKV in human moDC (C) and moMØ (D). The MOI for infection was 0.5 for moDC and

0.1 for moMØ. Gene quantities were normalized against human *actin* (*NM_001101.4*). The data are presented as the mean ± s.e.m. (C) $n = 6$ independent samples. (D) $n = 5$ (Donor 2) or $n = 6$ (Donor 1, Donor 3) independent samples. (E-I) Knockdown of *AalbVA-1* in *A. albopictus* impaired ZIKV transmission. (E-F) The *AalbVA-1* silencing efficiency was detected by qPCR (E) and western blotting (F) at 11 days post-gene silencing. (G) Silencing of *AalbVA-1* gene did not influence the ZIKV load in the mosquito salivary glands. The ZIKV burden was determined at 8 days post-infection. Each line represents the median value of the group. (E, G) Gene quantities were normalized against *A. albopictus actin* (*AALF010408*). (E) $n = 10$ (GFP) or $n = 9$ (*Aalb*VA-1) mosquito salivary glands per group. (G) $n = 16$ (GFP) or $n = 14$ (*Aalb*VA-1) mosquito salivary glands per group. A nonparametric Mann-Whitney test was used for the statistical analysis. (H) The ZIKV load in mice plasma was detected by a plaque assay ($n = 8$ mice per group). Each line represents the mean value of the group. (I) Animal mortality was recorded daily ($n = 8$ mice per group). (C-E, G-H) A nonparametric Mann-Whitney test was used for the statistical analysis. (I) Survival rates of the infected mice were statistically analysed using the log-rank (Mantel-Cox) test. *$p < 0.05$, **$p < 0.01$, *n.s.*, not significant ($p \geq 0.05$). (C-E, G-I) The data were combined based on 2 independent experiments. Source data are provided as a Source Data file.

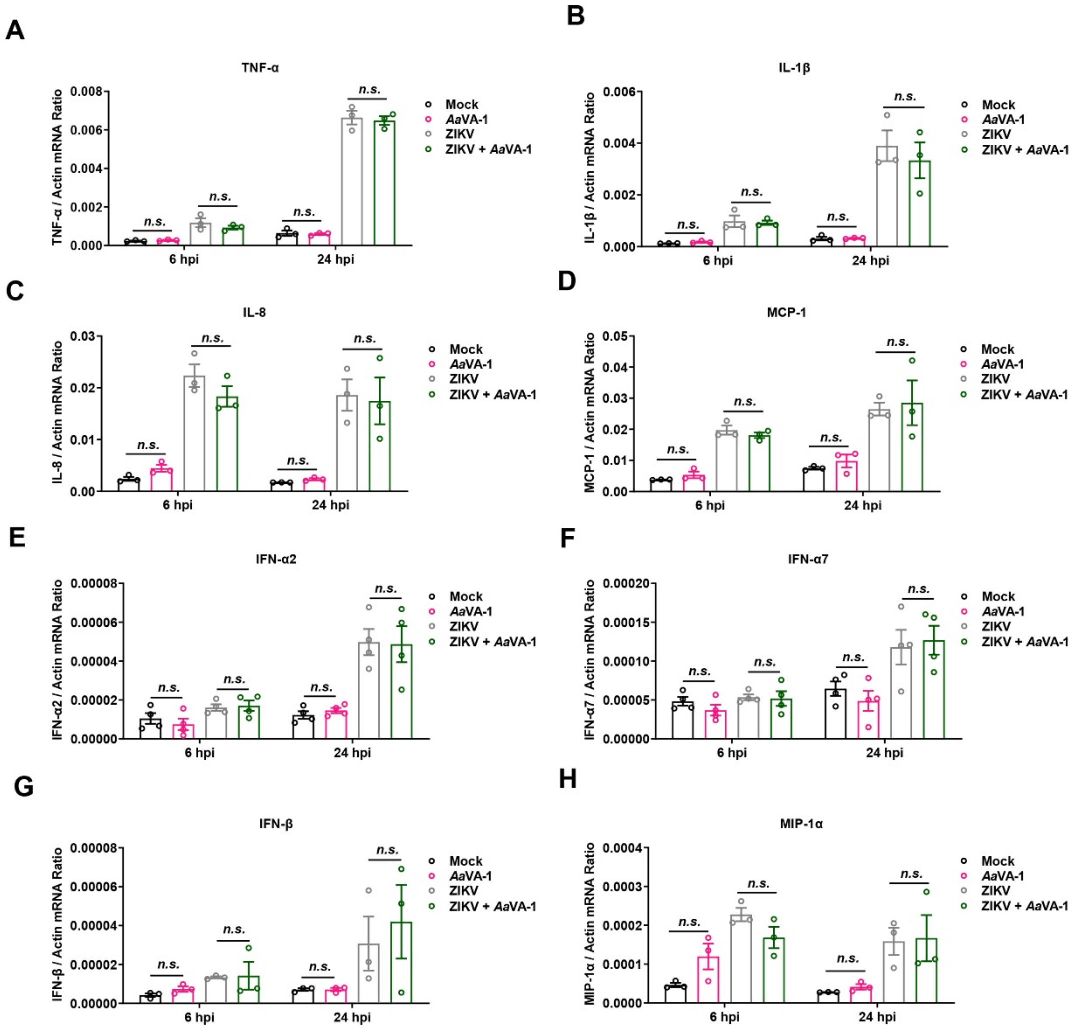

Supplementary Figure 7. Assessment of *Aa*VA-1-mediated cytokine regulation. *Aa*VA-1 (1 μg/ml) mixed with or without ZIKV (0.1 MOI) was used to infect THP-1 cells. An equal amount of BSA served as a mock control. The cells were collected at 6 and 24 h post-infection (hpi) to detect the expression of TNF-α (A), IL-1β (B), IL-8 (C), MCP-1 (D), IFN-α2 (E), IFN-α7 (F), IFN-β (G) and MIP-1α (H) by qRT-PCR. $n = 3$ (A-D, G-H) or $n = 4$ (E-F) independent samples. The data are presented as the mean ± s.e.m. A nonparametric Mann-Whitney test was used for the statistical analysis. *n.s.*, not significant ($p \geq 0.05$). The experiments were repeated 2 times with the similar results. Source data are provided as a Source Data file.

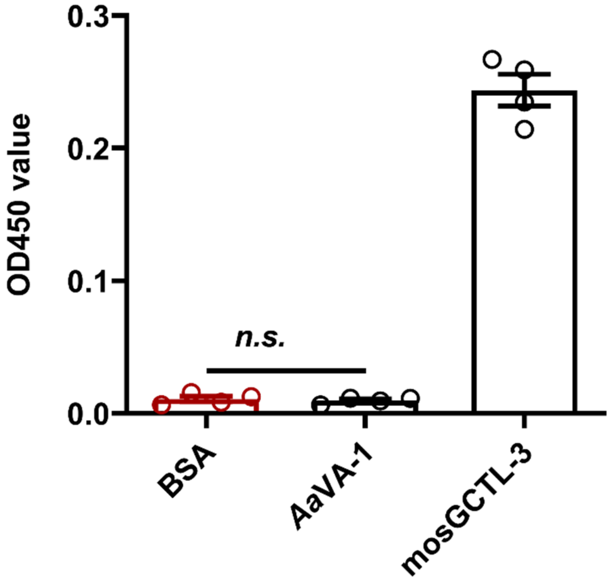

Supplementary Figure 8. *Aa*VA-1 cannot directly interact with DENV. The purified *Aa*VA-1 was mixed with DENV E protein for an ELISA assay. An equal amount of BSA served as a negative control. mosGCTL-3, which can directly bind the envelop protein of DENV, was used as a positive control. Binding was probed using an anti-V5 antibody. $n = 4$ independent samples. The data are presented as the mean ± s.e.m. A nonparametric Mann-Whitney test was used for the statistical analysis. *n.s.*, not significant ($p \geq 0.05$). The experiment was repeated 3 times with the similar results. Source data are provided as a Source Data file.

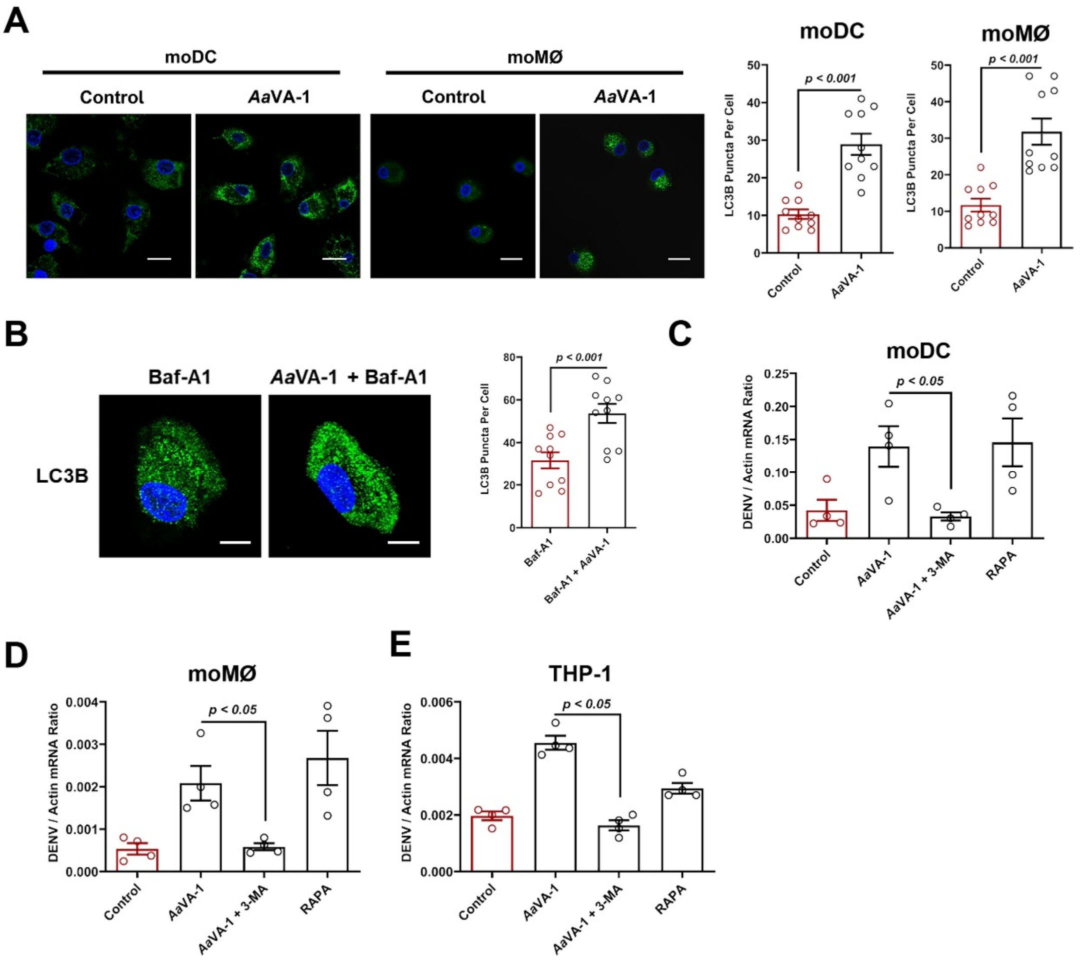

Supplementary Figure 9. 3-MA-mediated interruption of autophagy impaired the *Aa*VA-1-mediated DENV enhancement in human monocyte-lineage immune cells. (A) *Aa*VA-1-mediated activation of autophagy in human moDC and moMØ cells. *Aa*VA-1 (1 μg/ml) was incubated with moDC and moMØ cells for 6 h. An equal amount of BSA served as a mock control. At 6 h post-incubation, the cells were stained with anti-LC3B antibody, and the nuclei were stained blue with DAPI (left panel). Images were examined by Zeiss LSM 780 meta confocal microscopy. Scale bars, 20 μm. LC3B-posivitive puncta were quantified in the right panels. n = 10 cells per group. (B) *Aa*VA-1 (1 μg/ml) was incubated with moMØ cells with or without Baf-A1. At 6 h post-incubation, the cells were stained with anti-LC3B antibody, and the nuclei were stained blue with DAPI (left panel). Images were examined by Zeiss LSM 780 meta confocal microscopy. Scale bars, 10 μm. LC3B-posivitive puncta were quantified in the right panels. n = 10 cells per group. (C-E) *Aa*VA-1 (1 μg/ml) with or without 5 mM 3-MA was incubated with human moDC (C), moMØ (D) and THP-1 cells (E). An equal amount of BSA served as a mock control. The autophagy agonist RAPA

was used as a positive control. At 24 h post-incubation, the viral load was detected by qRT-PCR. Gene quantities were normalized against human *actin* (*NM_001101.4*). n = 4 independent samples. The data are presented as the mean ± s.e.m. A nonparametric Mann-Whitney test was used for the statistical analysis. The experiments were repeated 3 times (A-B) or 2 times (C-E) with the similar results. Source data are provided as a Source Data file.

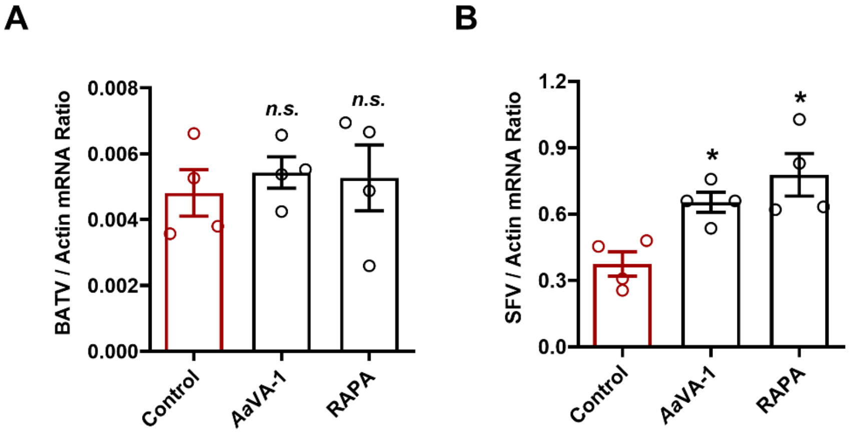

Supplementary Figure 10. The roles of *Aa*VA-1 in BATV and SFV infection. Human THP-1 cells were incubated with *Aa*VA-1 (1 μg/ml) and BATV (or SFV). The viral loads were detected at 24 h post-infection by qRT-PCR. BSA was incubated with the viruses as a mock control. The qPCR primers for BATV S gene and SFV nsP1 gene were listed in Supplementary Table 4. Gene quantities were normalized against human *actin* (*NM_001101.4*). n = 4 independent samples. The data are presented as the mean ± s.e.m. A nonparametric Mann-Whitney test was used for the statistical analysis. *$p < 0.05$, *n.s.*, not significant ($p \geq 0.05$). The experiments were reproduced 2 times with the similar results. Source data are provided as a Source Data file.

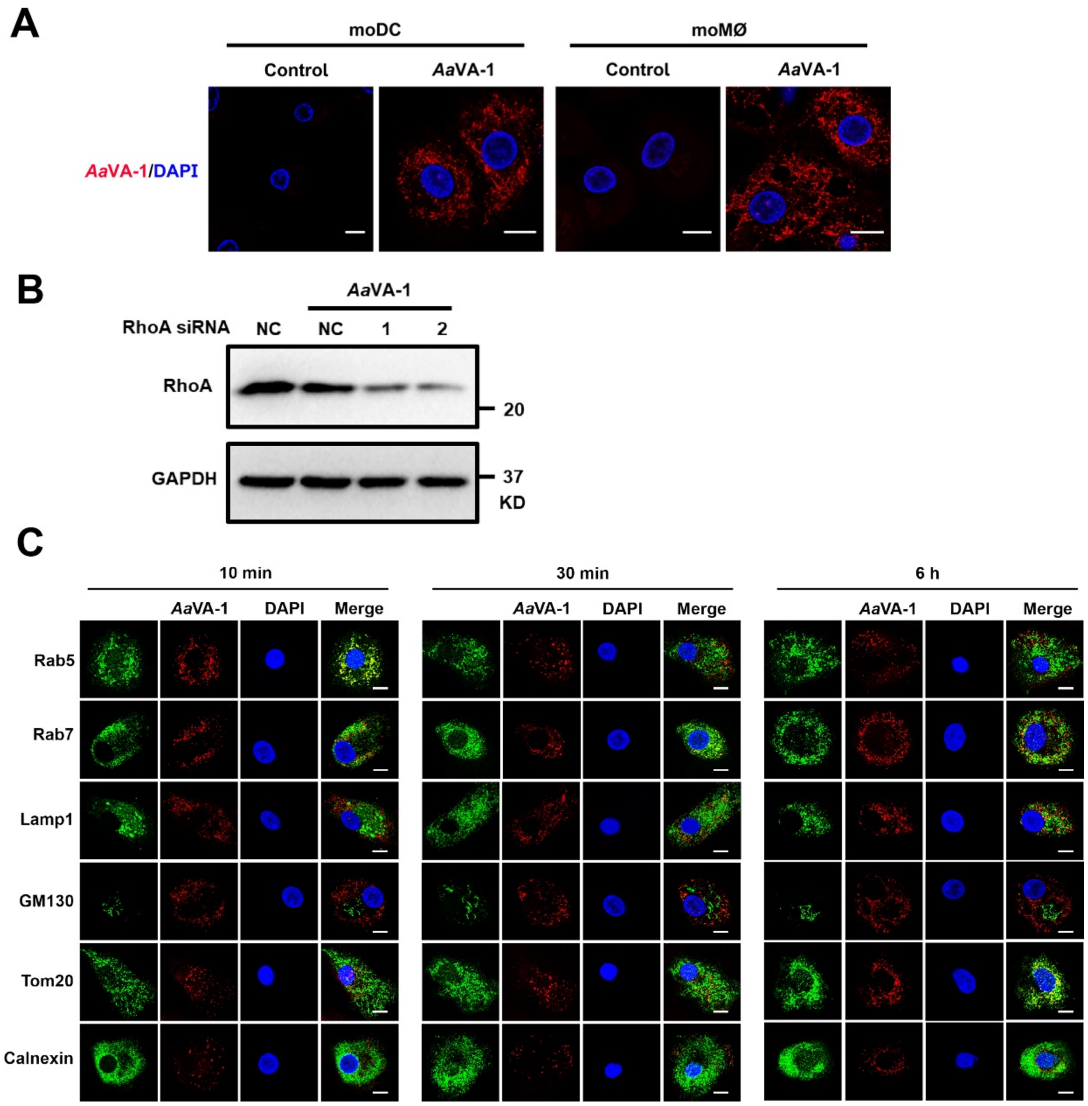

Supplementary Figure 11. Localization of *Aa*VA-1 in human immune cells. (A) The localization of *Aa*VA-1 in human moDC and moMØ cells. Purified *Aa*VA-1 (5 μg/ml) was incubated with human moDC and moMØ cells for 6 h. *Aa*VA-1 was stained with anti-mouse IgG Alexa-546 (red). Nuclei were stained blue with DAPI. (B) siRNA-mediated silencing of the *RhoA* gene in human THP-1 cells. The expression of RhoA was assessed by Western blotting. GAPDH served as an internal control. (C) The intracellular localization of *Aa*VA-1 after endocytosis. The human moMØ cells were incubated with *Aa*VA-1 (red), and then the cells were collected to stain the cellular organelles (green), such as early endosome (Rab5), late endosome (Rab7), Golgi (GM130), endoplasmic reticulum (Calnexin), lysosome (Lamp1), and mitochondria (Tom20), in a time course post incubation. Nuclei were stained blue with DAPI. Images were examined using a Zeiss LSM 780 meta confocal microscope. Scale bars, 10 μm. The experiments were repeated 3 times (A-B) or 2 times (C) with the

similar results.

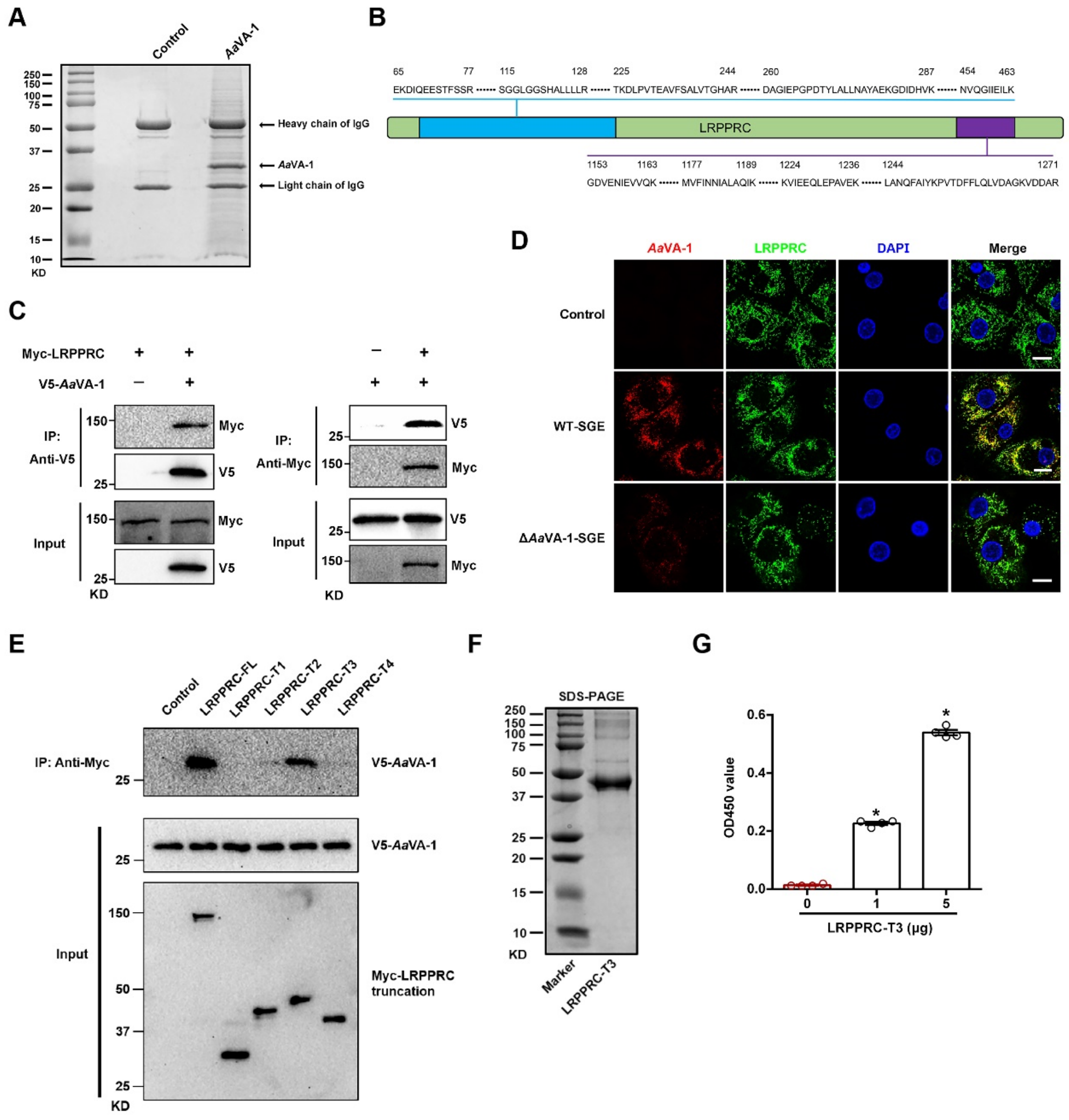

Supplementary Figure 12. Interaction between *Aa*VA-1 and LRPPRC. (A-B) Two micrograms of *Aa*VA-1 purified protein was incubated with the THP-1 cell lysates for a pull-down assay. The collected proteins were separated by SDS-PAGE (A). The protein gels were subsequently characterized by LC-MS. (B) Schematic representation of LRPPRC identified by LC-MS. The sequences of the identified peptides are presented in the figure. (C) *Aa*VA-1 interacted with LRPPRC in a co-IP assay. Two micrograms of purified *Aa*VA-1 (V5 tag) was incubated with lysates from LRPPRC (Myc tag)-expressing cells to investigate the protein interactions. The protein complex was pulled down with either anti-V5 or anti-Myc antibodies and detected by the other antibody. (D) Co-localization of *Aa*VA-1 and LRPPRC in SGE-incubated human moMØ cells. The cells were infected with ZIKV (0.1 MOI) in combination with either Δ*Aa*VA-1-SGE or WT-SGE. After 6 h of incubation, *Aa*VA-1 was

stained with anti-mouse IgG Alexa-546 (red), and LRPPRC was stained with anti-rabbit IgG Alexa-488 (green). Nuclei were stained blue with DAPI. Images were examined using a Zeiss LSM 780 meta confocal microscope in multi-track mode. Scale bars, 10 μm. (E) The interaction between *Aa*VA-1 and LRPPRC truncations. Four truncations of LRPPRC (T1: 1-337 aa; T2: 338-711 aa; T3: 712-1067 aa; T4: 1068-1394 aa; FL: full length) were constructed and expressed in human 293T cells. The cell lysates were mixed with 2 μg *Aa*VA-1 for an immunoprecipitation assay. (F) Expression and purification of LRPPRC-T3 peptide using a 293F expression system. The *LRPPRC-T3* gene was cloned into the pcDNA3.1/Myc-His C expression vector. Recombinant LRPPRC-T3 peptide was expressed and purified using a Cobalt-His column. (G) LRPPRC-T3 peptide directly binds *Aa*VA-1. The interaction was determined by ELISA. The commercial peroxidase substrate system was used for signaling detection, and the optical density (OD) at 450 nm was measured with an ELISA reader. n = 4 independent samples. The data are presented as the mean ± s.e.m. A nonparametric Mann-Whitney test was used for statistical analysis. The experiments were repeated 3 times with the similar results. Source data are provided as a Source Data file.

A

Human PPR protein	Accession number
LRPPRC	NM_133259.4
POLRMT	NM_005035.4
MRPS27	NM_015084.3
PTCD1	NM_015545.4
PTCD2	NM_024754.5
PTCD3	NM_017952.6
MRPP3	NM_014672.4

B

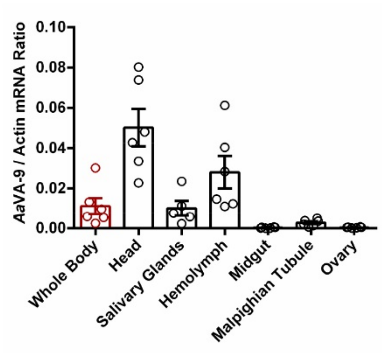

C **D**

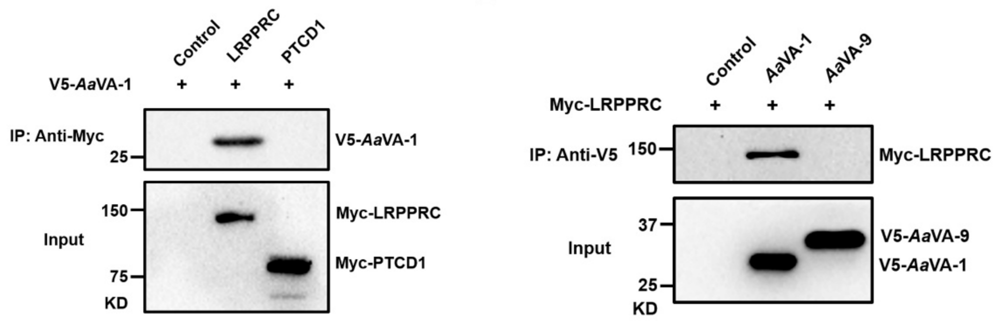

Supplementary Figure 13. Binding specificity between human LRPPRC and *Aa*VA-1. (A) Human proteins with PPR domains. (B) Expression of *Aa*VA-9 in various tissues of *A. aegypti*. Gene quantities were normalized against *A. aegypti actin* (*AAEL01119*). The data are presented as the mean ± s.e.m. A nonparametric Mann-Whitney test was used for statistical analysis. *n* = 5-6 independent samples. (C) Assessment of the interaction between *Aa*VA-1 and PTCD1. (D) Investigation of the interaction between *Aa*VA-9 and LRPPRC. *Aa*VA-1 and *Aa*VA-9 were expressed in the conditioned supernatants of *Drosophila* S2 cells, while human LRPPRC and PTCD1 were ectopically generated in human 293T cells. The interaction was assessed by an immunoprecipitation assay. The experiments were repeated 2 times with the similar results. Source data are provided as a Source Data file.

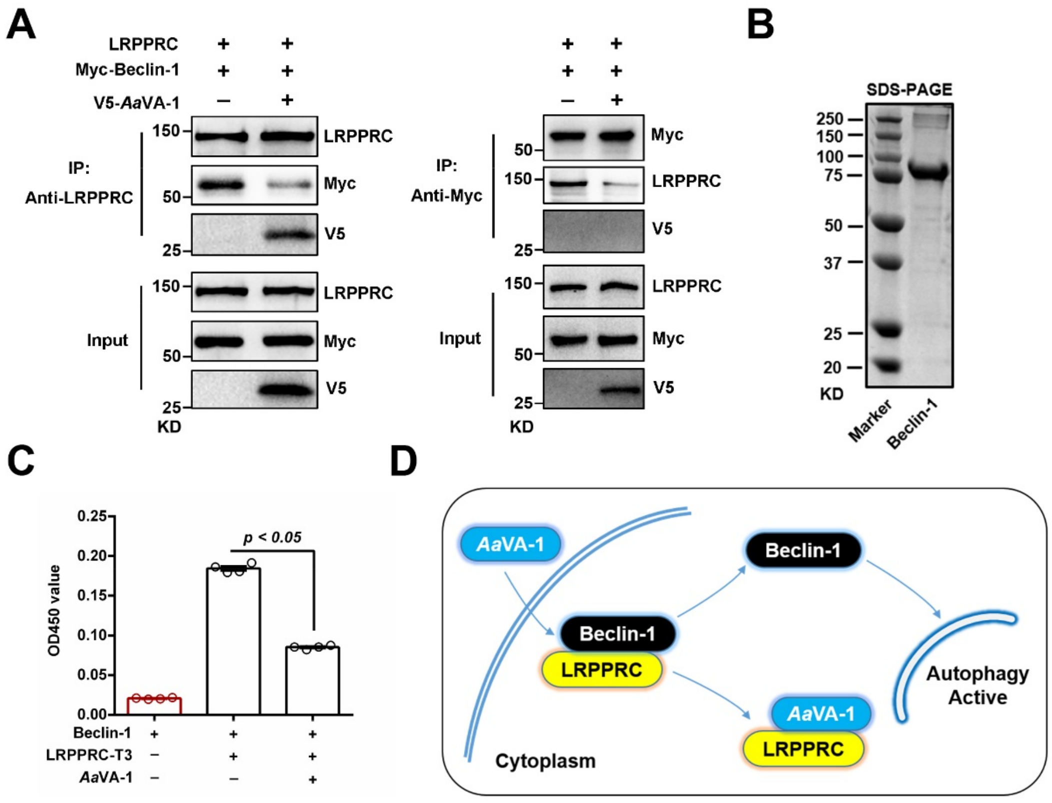

Supplementary Figure 14. *Aa*VA-1 liberates Beclin-1 from the LRPPRC-inhibition state. (A) *Aa*VA-1 impaired the binding between LRPPRC and Beclin-1. The purified *Aa*VA-1 (V5 tag) was incubated with lysates from LRPPRC/Beclin-1 (Myc tag)-expressing cells to investigate the protein interactions. The protein complex was pulled down with either anti-LRPPRC or anti-Myc antibodies. (B) Expression and purification of Beclin-1 from *E. coli*. The *Beclin-1* gene was cloned into the pGEX-6p-2 expression vector. Recombinant Beclin-1 (GST tag) was expressed and purified using glutathione-sepharose. (C) Incubation of *Aa*VA-1 impaired the interaction between Beclin-1 and LRPPRC-T3. Three purified proteins were used in this experiment. The interaction was assessed by ELISA. A commercial peroxidase substrate system was used for signaling detection, and the optical density (OD) at 450 nm was measured with an ELISA reader. n = 4 independent samples. The data are presented as the mean ± s.e.m. A nonparametric Mann-Whitney test was used for the statistical analysis. (D) Schematic representation of *Aa*VA-1-mediated activation of autophagy. *Aa*VA-1 acts as a competitor to release Beclin-1 from LRPPRC-mediated sequestration, thereby enabling the initialization of downstream autophagy signaling. (A-C)

The experiments were repeated 3 times with the similar results. Source data are provided as a Source Data file.

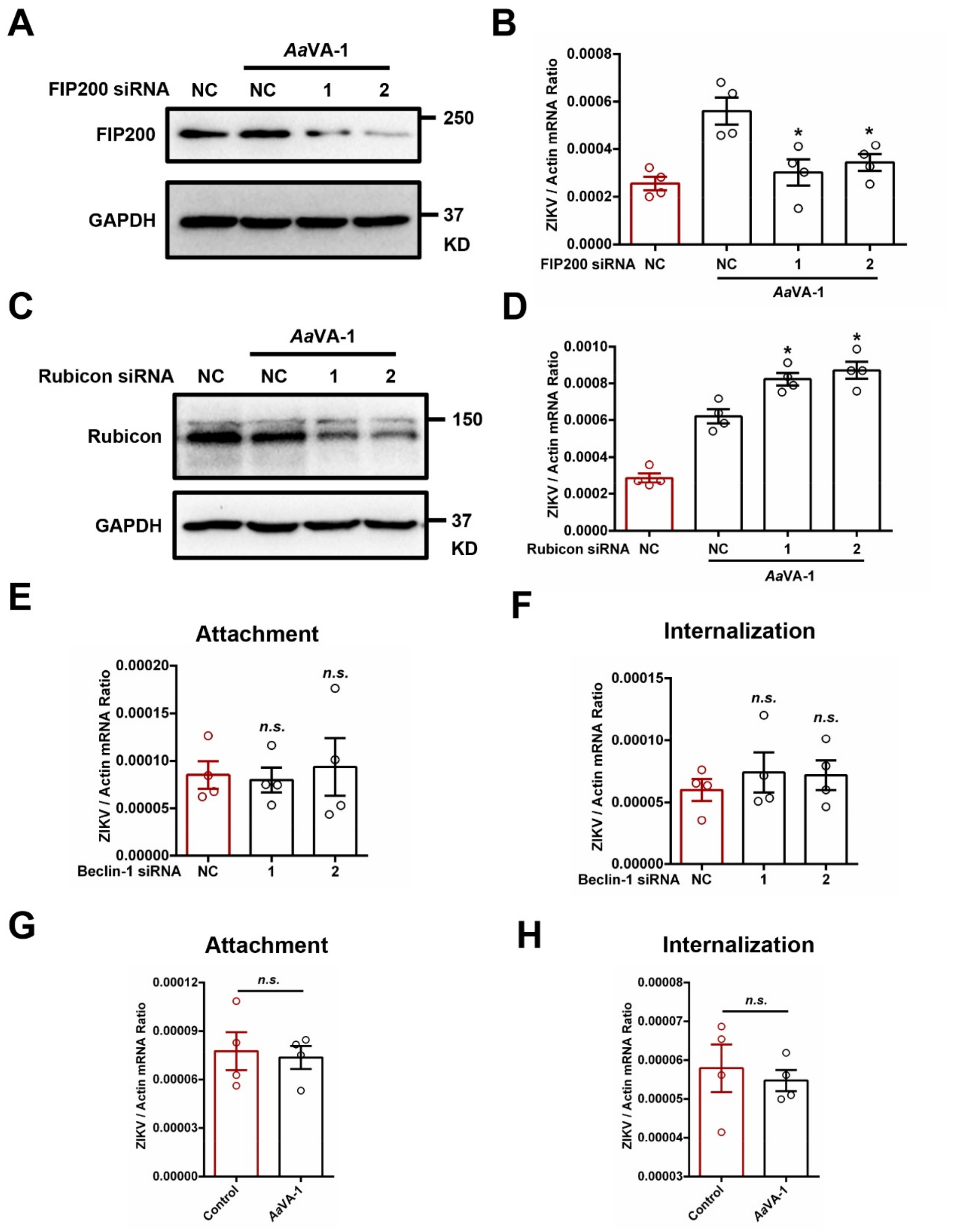

Supplementary Figure 15. *Aa*VA-1-mediated released Beclin-1 does not participate in the LAP process.

(A-D) siRNA-mediated knockdown of *FIP200*, but not *Rubicon*, reduced ZIKV infection in the *Aa*VA-1-incubated THP-1 cells. (A and C) siRNA-mediated silencing of *FIP200* (A) and *Rubicon* (C) in human THP-1 cells. The degree of silencing was measured by Western

blotting. GAPDH served as an internal control. (B and D) Suppression of *FIP200* (B), but not *Rubicon* (D), reduced ZIKV infection in *Aa*VA-1-incubated THP-1 cells.

(E-F) Beclin-1 did not regulate ZIKV entry. (E) ZIKV (0.1 MOI) was incubated with either *Beclin-1* silenced or mock THP-1 cells at 4 °C. After stringent washes by cold PBS, the attached ZIKV particles were quantified by qRT-PCR. (F) ZIKV was incubated with either the *Beclin-1*-silenced THP-1 cells or the control siRNA-treated cells at 37 °C for 2 h, and ZIKV burden was quantified by qRT-PCR.

(G-H) Assessment of ZIKV attachment and internalization in THP-1 cells with or without *Aa*VA-1 incubation. ZIKV (0.1 MOI) and 1 μg/ml *Aa*VA-1 were premixed to incubate with human THP-1 cells at 4 °C (G) and 37 °C (H). After stringent washes, the attached ZIKV particles were detected by qRT-PCR.

(B, D, E-F, G-H) Gene quantities were normalized against human *actin* (*NM_001101.4*). $n = 4$ independent samples. The data are presented as the mean ± s.e.m. A nonparametric Mann-Whitney test was used for the statistical analysis. *$p < 0.05$. *n.s.*, not significant ($p \geq 0.05$).

(A-H) The experiments were repeated 2 times with the similar results. Source data are provided as a Source Data file.

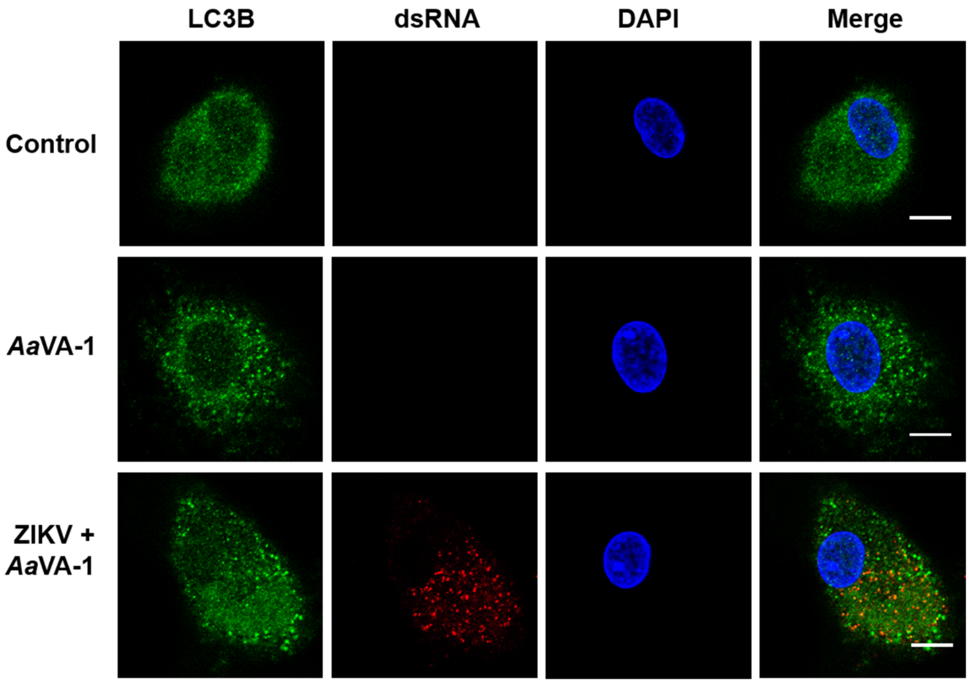

Supplementary Figure 16. The ZIKV replication compartment did not co-localize with the LC3B-positive puncta in *Aa*VA-1-incubated macrophages. *Aa*VA-1 (1 μg/ml) and ZIKV (0.1 MOI) were incubated with moMØ. After 24 h of infection, the cells were stained with LC3B (green) antibody. ZIKV dsRNA, which represents the viral replication compartment, was stained by anti-dsRNA J2 (red). Nuclei were stained blue with DAPI. Images were examined using a Zeiss LSM 780 meta confocal microscope in multi-track mode. Scale bars, 10 μm. The experiments were repeated 2 times with the similar results.

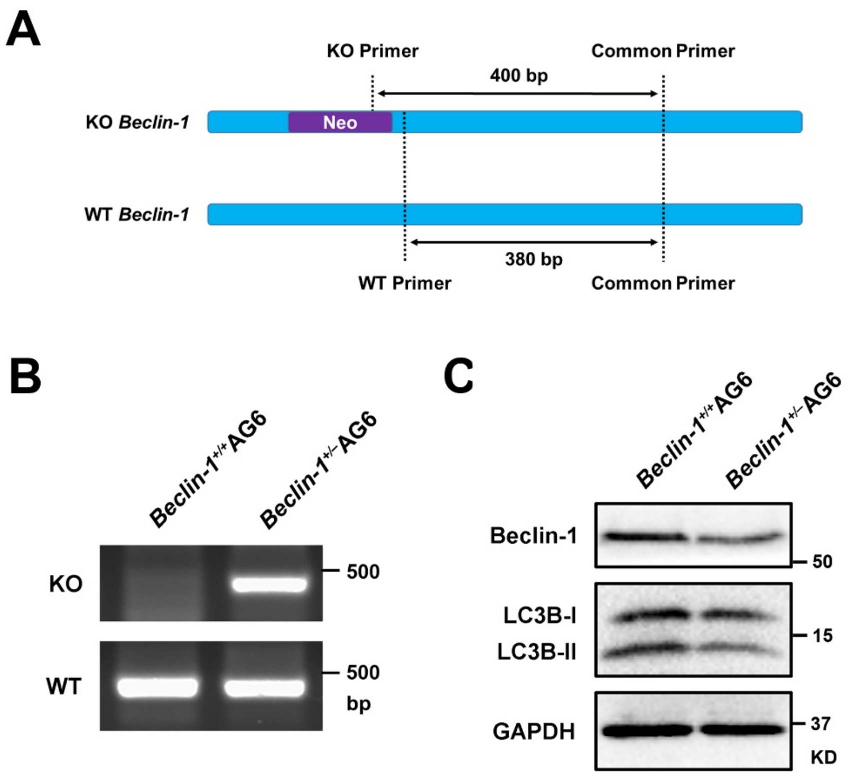

Supplementary Figure 17. Generation of a *Beclin-1*$^{+/-}$AG6 mouse strain. (A) Schematic representation of PCR genotyping. The targeting construct contains a cassette with the neomycin resistance gene (Neo) that has replaced exons 1 and 2 of the *Beclin-1* gene. (B) Mouse genotyping by PCR. The mouse tail was collected for PCR. The primers are presented in Supplementary Table 4. (C) Assessment of Beclin-1 protein expression and autophagy in the *Beclin-1*$^{+/-}$AG6 mouse. After 48 h starvation, *Beclin-1*$^{+/-}$AG6 lung samples were used to western blotting analysis with anti-Beclin-1 and anti-LC3B antibodies. The *Beclin-1*$^{+/+}$AG6 mouse was used as a mock control. (B-C) The experiments were repeated 3 times with the similar results.

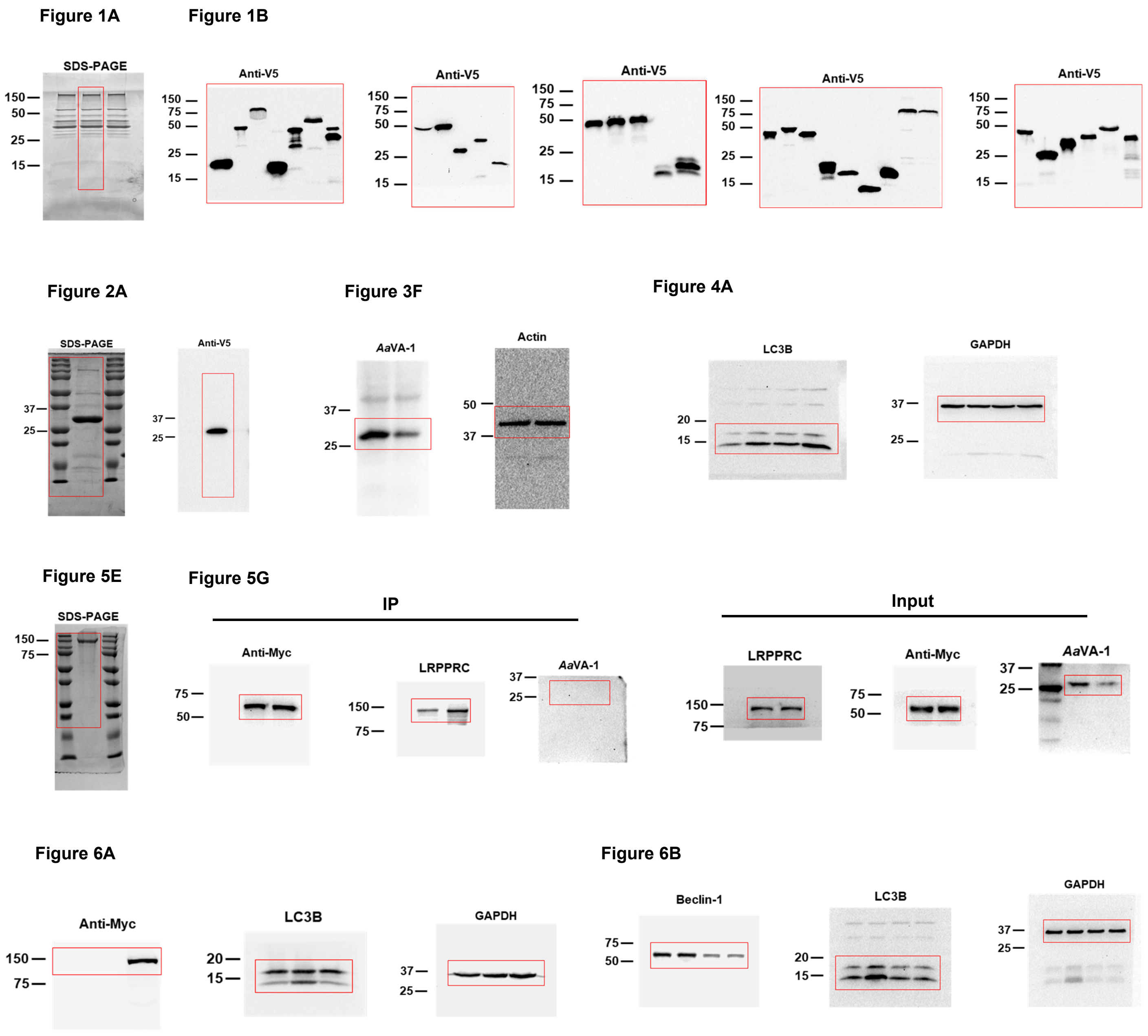

Supplementary Figure 18. Uncropped blots/gels for Figures 1-6.

Supplementary Figure 4

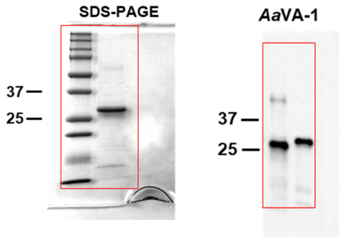

Supplementary Figure 6B

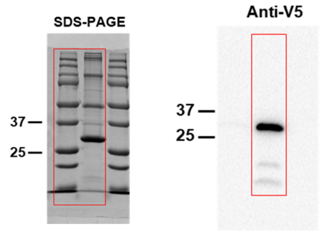

Supplementary Figure 6F

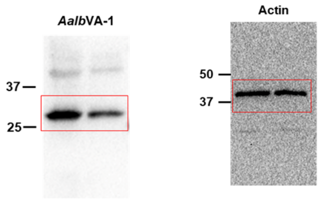

Supplementary Figure 11B

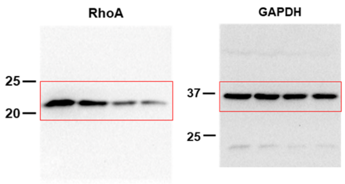

Supplementary Figure 12A

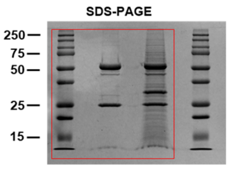

Supplementary Figure 12C (left panel)

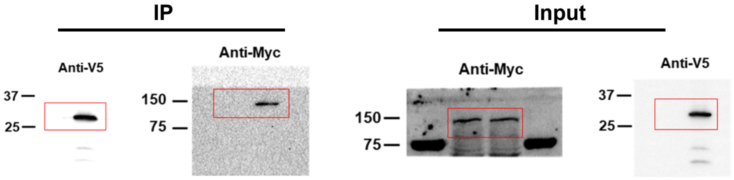

Supplementary Figure 12C (right panel)

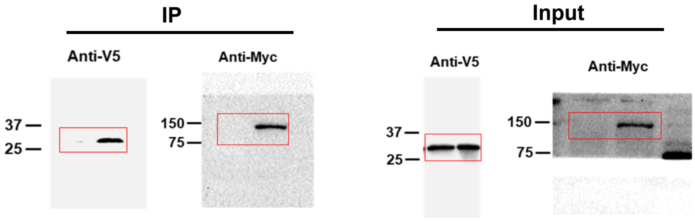

Supplementary Figure 12E

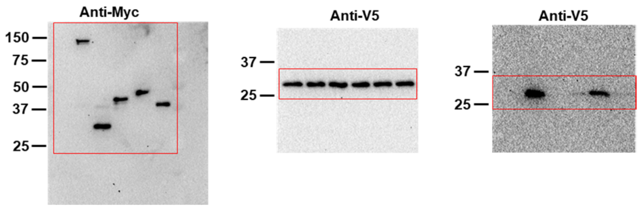

Supplementary Figure 12F

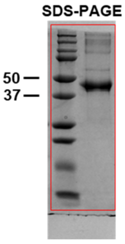

Supplementary Figure 13C

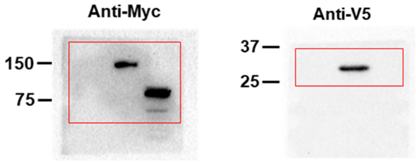

Supplementary Figure 13D

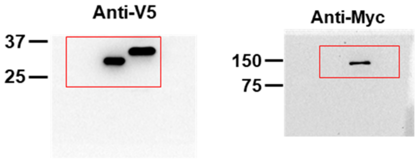

Supplementary Figure 19. Uncropped blots/gels for Supplementary Figures 4, 6, 11, 12, 13.

Supplementary Figure 14A (left panel)

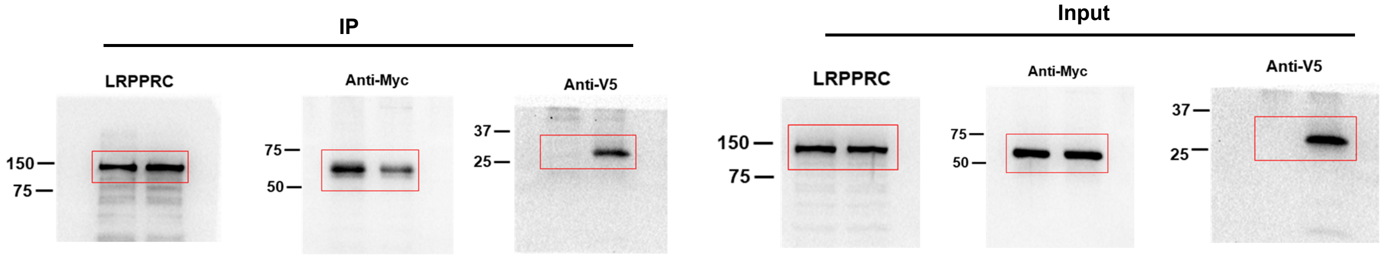

Supplementary Figure 14A (right panel)

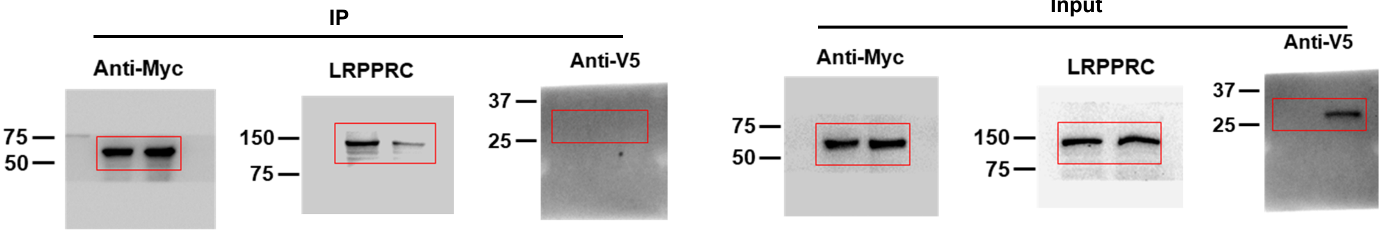

Supplementary Figure 14B

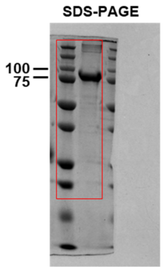

Supplementary Figure 15A

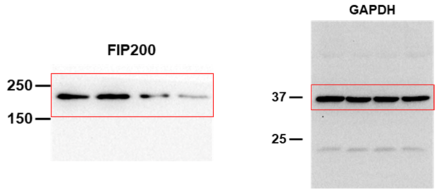

Supplementary Figure 15C

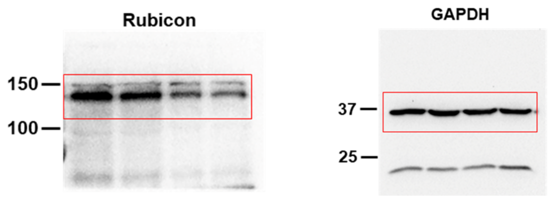

Supplementary Figure 17B

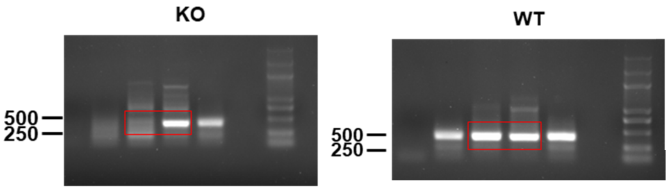

Supplementary Figure 17C

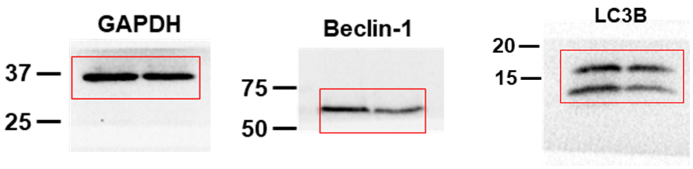

Supplementary Figure 20. Uncropped blots/gels for Supplementary Figures 14, 15, 17.

Supplementary Table 1 Identified proteins in *A. aegypti* saliva by LC-MS/MS

Gene Number	Score	Description
AAEL006347	8418.65	Apyrase Precursor
AAEL006485	2413.71	Inosine-uridine preferring nucleoside hydrolase
AAEL006424	2262.26	37 kDa salivary gland allergen Aed a 2 Precursor
AAEL003182	2202.37	Serine Protease Inhibitor (serpin) homologue
AAEL000749	1852.42	Conserved hypothetical protein
AAEL006417	1468.68	D7 protein, putative
AAEL010235	1264.15	30 kDa salivary gland allergen Aed a 3 Precursor
AAEL000726	1260.68	Fibrinogen and fibronectin
AAEL000556	1247.66	C-Type Lectin (CTL)
AAEL006096	1049.90	Gelsolin precursor
AAEL002704	922.50	Serine Protease Inhibitor (serpin) homologue
AAEL005672	878.16	Adenosine deaminase
AAEL007420	865.74	Serine Protease Inhibitor (serpin) homologue
AAEL000533	865.43	C-Type Lectin (CTL)
AAEL000748	801.34	Conserved hypothetical protein
AAEL010228	736.52	Conserved hypothetical protein
AAEL000732	585.68	Conserved hypothetical protein
AAEL003600	425.26	Conserved hypothetical protein
AAEL000793	417.39	Venom allergen
AAEL003053	416.16	Allergen, putative
AAEL003601	376.62	Conserved hypothetical protein
AAEL013532	329.88	Hypothetical protein
AAEL003585	243.14	Conserved hypothetical protein
AAEL009524	231.66	Alpha-amylase
AAEL000392	230.17	Probable maltase Precursor
AAEL009852	182.64	Conserved hypothetical protein
AAEL013029	138.92	Deoxyribonuclease I, putative
AAEL011510	113.65	Multiple inositol polyphosphate phosphatase
AAEL009081	106.93	Conserved hypothetical protein
AAEL003057	102.74	Allergen, putative
AAEL008305	82.93	Conserved hypothetical protein
AAEL007986	78.71	Conserved hypothetical protein
AAEL002693	78.02	Venom allergen
AAEL006423	75.81	Conserved hypothetical protein
AAEL009670	75.38	C-Type Lysozyme (Lys-D)
AAEL003584	52.68	Hypothetical protein
AAEL007064	52.19	Gram-Negative Binding Protein (GNBP)
AAEL006406	47.87	Conserved hypothetical protein
AAEL018258	45.45	Hypothetical protein
AAEL007394	42.91	Conserved hypothetical protein
AAEL003107	40.78	Conserved hypothetical protein
AAEL006351	27.61	Conserved hypothetical protein
AAEL003100	24.42	Conserved hypothetical protein
AAEL006380	18.95	Sphingomyelin phosphodiesterase
AAEL001163	15.74	Macroglobulin/complement
AAEL008876	14.82	Deoxyribonuclease I, putative
AAEL008274	11.21	Conserved hypothetical protein
AAEL002656	10.37	Conserved hypothetical protein
AAEL010242	10.21	Conserved hypothetical protein
AAEL008789	7.88	Apolipophorin-III, putative
AAEL004873	7.79	Conserved hypothetical protein
AAEL000575	7.75	Apyrase, putative
AAEL006408	7.70	Conserved hypothetical protein
AAEL004899	5.79	Hypothetical protein
AAEL007385	5.49	Ferritin subunit Precursor
AAEL004460	5.35	Sulfatase
AAEL008620	4.47	D7 protein, putative
AAEL002158	4.22	Secreted ferritin G subunit precursor, putative
AAEL002679	4.20	Hypothetical protein
AAEL001020	4.18	Anterior fat body protein
AAEL008364	4.00	Serine Protease Inhibitor (serpin)
AAEL007776	3.85	Conserved hypothetical protein
AAEL005596	3.77	Trypsin-epsilon, putative
AAEL001511	3.14	Conserved hypothetical protein
AAEL001641	2.69	Deoxyribonuclease I, putative
AAEL001964	1.91	Protein serine/threonine kinase, putative
AAEL007383	1.86	Secreted ferritin G subunit precursor, putative
AAEL006568	1.84	Serine protease
AAEL001469	1.80	Kek1
AAEL004522	1.77	Gambicin anti-microbial peptide
AAEL000019	1.61	Conserved hypothetical protein

Score > 25

Supplementary Table 2 *Aa*VA-1 paralogues in *A. aegypti*

Gene Name	Gene Number	Length (AA)	Identity with *Aa*VA-1
*Aa*VA-1	AAEL000793	255	/
*Aa*VA-2	AAEL009239	258	60 %
*Aa*VA-3	AAEL013406	256	43 %
*Aa*VA-4	AAEL006524	260	40 %
*Aa*VA-5	AAEL002476	262	43 %
*Aa*VA-6	AAEL002682	260	40 %
*Aa*VA-7	AAEL002693	259	42 %
*Aa*VA-8	AAEL010269	265	35 %
*Aa*VA-9	AAEL009695	262	36 %
*Aa*VA-10	AAEL004199	258	33 %
*Aa*VA-11	AAEL019798	265	32 %
*Aa*VA-12	AAEL011798	251	32 %
*Aa*VA-13	AAEL011797	251	31 %
*Aa*VA-14	AAEL019797	265	32 %
*Aa*VA-15	AAEL004407	255	37 %
*Aa*VA-16	AAEL005531	359	38 %
*Aa*VA-17	AAEL003053	255	31 %
*Aa*VA-18	AAEL003057	261	34 %

Supplementary Table 3 Identified proteins as *Aa*VA-1 binding proteins by LC-MS/MS

Accession number	Name	Score	Description
NM_006372.5	SYNCRIP	163.34	Synaptotagmin binding cytoplasmic RNA interacting protein
NM_001011.4	RPS7	106.63	Ribosomal protein S7
NM_021141.4	XRCC5	80.82	X-ray repair cross complementing 5
NM_004728.4	DDX21	66.87	DExD-box helicase 21
NM_001357.5	DHX9	66.66	DExH-box helicase 9
NM_000981.4	RPL19	53.25	Ribosomal protein L19
NM_003134.6	SRP14	49.60	Signal recognition particle 14
NM_002388.6	MCM3	48.65	Minichromosome maintenance complex component 3
NM_000971.4	RPL7	47.37	Ribosomal protein L7
NM_006805.4	HNRNPA0	47.12	Heterogeneous nuclear ribonucleoprotein A0
NM_005066.3	SFPQ	46.12	Splicing factor proline and glutamine rich
NM_031370.3	HNRNPD	45.10	Heterogeneous nuclear ribonucleoprotein D
NM_005322.2	HIST1H1B	44.44	Histone cluster 1 H1 family member b
NM_006739.4	MCM5	44.01	Minichromosome maintenance complex component 5
NM_012218.4	ILF3	42.14	Interleukin enhancer binding factor 3
NM_006275.6	SRSF6	41.01	Serine and arginine rich splicing factor 6
NM_002911.4	UPF1	39.02	UPF1, RNA helicase and ATPase
NM_001018.4	RPS15	38.52	Ribosomal protein S15
NM_005594.6	NACA	36.96	Nascent polypeptide-associated complex alpha subunit
NM_004515.4	ILF2	36.95	Interleukin enhancer binding factor 2
NM_133259.4	LRPPRC	35.29	Leucine rich pentatricopeptide repeat containing
NM_003142.5	SSB	35.25	Sjogren syndrome antigen B
NM_000661.4	RPL9	34.92	Ribosomal protein L9
NM_000994.4	RPL32	33.73	Ribosomal protein L32
NM_018715.4	RCC2	33.67	Regulator of chromosome condensation 2
NM_000989.4	RPL30	32.88	Ribosomal protein L30
NM_007007.3	CPSF6	32.82	Cleavage and polyadenylation specific factor 6

Supplementary Table 4 Primers and siRNA

Primers for cloning into pET28a (+)	Upper primer	Lower primer
*Aa*VA-1	CTCGGATCCAACTACTGCGACCAATCTCT	ATACTCGAGTTAGCCTCTGTACGATTCCG
**Primers for cloning into pGEX-6P-2**	**Upper primer**	**Lower primer**
Beclin-1	AGGGGCCCCTGGGATCCATGGAAGGGTCTAAGAC	CAGTCAGTCACGATGCGGCCGCTCATTTGTTATAAAATTGTGAGGACACCC
**Primers for cloning into pMT/BiP/V5-His A**	**Upper primer**	**Lower primer**
AAEL006347	TCACCGGGTACCTACGGATAATATGCCC	TCCCGGCTCGAGAGTACATTTCGAACC
AAEL006485	CGCGCCATGGCAAAGTCCAGCAACA	GGCGCGCTCTAGACGATTTATTCCAAGC
AAEL006424	GGGTACCTGAGTCCGACGTCGA	CTCGAGTGCCTTTGCGACGCC
AAEL003182	GCGGGGTACCTAAACCCACTCAAGGT	GCCGCTCGAGACCCCTATCATTCCA
AAEL000749	CGGCGGGGTACCTGATGAAAATCACTCA	GGCCCGCTCGAGATTTTTCGGTCGTAT
AAEL006417	CGGGTACCTGCGGTGATTGCAAGG	CGCCGCTCGAGAGTACATTTCGAACC
AAEL010235	TAATACGGGTACCTAGGCCCATGCCCGAAG	GTCCGGCTCGAGACGTCCTTTGGATGA
AAEL000726	GCCTAGCCATGGGAGGAAGACAATCCT	GGCCGAGTCTAGATTCAGCCGTTTTCAAG
AAEL000556	TACCCGGGTACCTCAGCAGAAGTGTGAT	GACCCGGCTCGAGATTTTTCTTCCTCTC
AAEL006096	GGTACCTGGTACGCCACCCTCC	CCCTCGAGGTATCGCGGTGGCTT
AAEL002704	GCGCCCATGGTCTCAATTCACGTCG	CGCGCTCTAGACTGATAACCAACCGG
AAEL005672	CGGGGTACCTGCACCTTTATGGGAC	CGCCGCTCGAGACTACACTGGATCTT
AAEL007420	GGGGTACCTGTTCCTCTTCCCTGC	GCCGCTCGAGCCGATTCAGATCTTC
AAEL000533	TATTCCGGGTACCTCAGCAGACATGCGAC	TAATTAGGCTCGAGCGCCTGTTCGCACAC
AAEL010228	AATGCCATGGAGGCCAACTCCCGAAG	GCCGGCGTCTAGATGATCTATTGGAAGC
AAEL003600	CCCATGGAGGCCAACTCCCGAA	CGCGCGCTCTAGATGATCTATTGGAAGC
AAEL000793	TCACCGGGTACCTAACTACTGCGACCAAT	TAACGGCTCGAGGCCTCTGTACGATTC
AAEL003601	GCGGGGTACCTCAGTTTCTCTCACCA	GCGGCCGCCAGCTTACCTGTTGTG
AAEL013532	CGGGGTACCTGGTCGAAAGCTCAAC	GCCGCTCGAGAACAAGTCTGGATCC
AAEL009524	GCGGACTAGTTTGGACTGGTGGGAAC	GCCGCTCGAGAGAAACAATCGGGTTC
AAEL000392	GCGGACTAGTTTGGACTGGTGGGAACA	GCCGCTCGAGAGAAACAATCGGGTTCT
AAEL011510	GCGCGGGGTACCTAAAAGTAGCAGATGTT	CCGCTCGAGTCTGCAGTACATTTCGG
AAEL008305	GGGTACCTGAGTCCGACGTCGA	CTCGAGTGCCTTTGCGACGCC
AAEL007986	GCGCCGGGGTACCTAAAACTTTCTCAGAA	GCGCCGCTCGAGCATGCAATATGAAAG
AAEL002693	GCGGGGTACCTACAAACTACTGTGACC	CCGCTCGAGAGGATTGTTGCTCACTC
AAEL006423	GGCGCGGTACCTCTCGAAGTTTCTCA	GCGCGCCTCGAGCTTACAAAAGGGTTT
AAEL009670	GCGCGGGGTACCTGATGAAAATCACTCA	GCGCCGCTCGAGATTTTTCGGTCGTAT
AAEL003584	CGCGCCATGGCAAAGTCCAGCAACA	GGCGCGCTCTAGACGATTTATTCCAAGC
AAEL006406	CGCGGGGTACCTAATGCGGTATCACA	GCGGCCCGCTCGAGATTACATAGATTTTG
AAEL007394	GCGCCCATGGTCTCAATTCACGTCG	CGCGCTCTAGACTGATAACCAACCGG
AAEL003107	GCGGGGTACCTGTGGATATATTCTCCG	ATATATATCCTCGAGGAACACCCCCTCGTCC
AAEL006351	TCCCCGGGTACCTAAATTCGATACTGCG	TAACGGCTCGAGACCAGAAGTCCTGGT
AAEL009695	ATCTCCATGGCCCGGGGTACCTATACCGGACTATTGCGATC	CGAAGGGCCCTCTAGACTCGAGGATCTCCTTATCGATAACATC
**Primers for cloning into pcDNA3.1/Myc-His C**	**Upper primer**	**Lower primer**
LRPPRC	TATGGTACCATGGCAGCCCTGCTGAGA	CGCTCTAGACGAGAAGAGTTTTCCCTCAA
Beclin-1	ATAGGTACCATGGAAGGGTCTAAGACG	CGCTCTAGACGTTTGTTATAAAATTGTGA
LRPPRC-T1	ATATTGCGGCCGCATGGCAGCCCTGCTGAGATC	GCGCGGCCCCGCGGATCTGGAATATATCTTCTTT
LRPPRC-T2	CTAGTCCAGTGTGGTGGCGGCCGCATGGCAATGAACCTC ATTTTACTT	TTGATGCCATTTCGAACCGCGGAGTAACCATGTCGGATTCAT
LRPPRC-T3	CTAGTCCAGTGTGGTGGCGGCCGCATGGGTGGCTATGCAGCTTTAAT	GCTTCTGCATTGATGCCATTTCGAACCGCGGAGCATTAAACACAATGTTTTGCTC
LRPPRC-T4	ATTGCGGCCGCATGGAAACCTACAGCAATCTCAT	GCGCCGCGGAGAAGAGTTTTCCCTCAATT
PTCD1	CCAAGCTTATGGACTTCGTGAGACTCGC	ATATTCTAGACTCCTGCCCCCAAGGGCACAT
**Primers for double-strand RNA synthesis**	**Upper primer**	**Lower primer**
GFP dsRNA	TAATACGACTCACTATAGGGGTGAGCAAGGGCGAGGAG	TAATACGACTCACTATAGGGCATGATATAGACGTTGTGGCTGTT
*Aa*VA-1 dsRNA	TAATACGACTCACTATAGGGAAACACCCGCGAGTTCAA	TAATACGACTCACTATAGGGTGTACGATTCCGACCTGA
*Aalb*VA-1 dsRNA	TAATACGACTCACTATAGGGAATGCGCCGCAACAATTCG	TAATACGACTCACTATAGGGCTGCGTGAAGTGTCCGAT
AAEL002693 dsRNA	TAATACGACTCACTATAGGGAACTCCCCATATAGCTTGCA	TAATACGACTCACTATAGGGGGCCGGATATCTTGCAATTT
AAEL005672 dsRNA	TAATACGACTCACTATAGGGAAGAGTATCATTTTCTTGGAG	TAATACGACTCACTATAGGGTCTCCTTGGAAAACTTAAATT
AAEL006417 dsRNA	TAATACGACTCACTATAGGGAAGAACCCAAAAGCAGCCCT	TAATACGACTCACTATAGGGTTTCTTTGCGGGCTACCTTT
**Primers for SYBR Green qRT-PCR**	**Upper primer**	**Lower primer**
ZIKV Envelope gene	CCGCTGCCCAACACAAG	CCACTAACGTTCTTTTGCAGACAT
DENV Envelope gene	CATTCCAAGTGAGAATCTCTTTGTCA	CAGATCTCTGATGAATAACCAACG
Human Actin	TGACGTGGACATCCGCAAAG	CTGGAAGGTGGACAGCGAGG
Aedes albopictus Actin	CGGAAGAGCACCCAGTTCTC	TGTGTCATCTTCTCGCGGTTAG
Aedes aegypti Actin	GAACACCCAGTCCTGCTGACA	TGCGTCATCTTCTCACGGTTAG
*Aa*VA-1	ACTCTCGTATGGGACGACGAA	CGACATTTATCGTGCGCAAA
*Aalb*VA-1	CGGCGAGCCCATTTACAC	TGCAAAGCCCCTTGAACTG
TNF-α	TCTTCTCGAACCCCGAGTGA	CCTCTGATGGCACCACCAG
MIP-1α	TGTTTGTGATTGTTTGCTCTGAGA	TCACCAGACGCGGTGTGA
IL-1β	TCTTCTCGAACCCCGAGTGA	CCTCTGATGGCACCACCAG
IL-8	CTCTTGGCAGCCTTCCTGATT	TATGCACTGACATCTAAGTTCTTTAGCA
MCP-1	CGCCTCCAGCATGAAAGTCT	GGAATGAAGGTGGCTGCTATG
IFN-β	CCAACAAGTGTCTCCTCCAAATT	GTAGGAATCCAAGCAAGTTGTAGCT
IFN-α2	GCTTGGGATGAGACCCTCCTA	CCCACCCCCTGTATCACAC
IFN-α7	AGGGCCTTGATACTCCTGG	TCCTCCTCCGGGAATCTGAAT
*Aa*VA-9	CTATTGCGATCCCCTGCTGT	ATTGGCCAGTTCGCTTCTCA
AAEL002693	ATCGTACCAGCAGAATAG	CGGTTGATTGATGATGTT
AAEL005672	TACTACGAAGAGACAGAGGAAT	AACCGACCAGGAGCATAA
AAEL006417	TGTGGAATTAGGAAGTATCAGA	CCAGTTCATTCTTGCTTGT
SFV nsP1 gene	CGTATTGGATTGGGTTTGACAC	TGCTGCACACAGTCCTATGTTC
BATV S gene	CAGTTCCAGACGATGGTCTTACC	GGTCACTCACTTTCAGAATCTTCTCA
**siRNA**	**Sense**	**Antisense**
Negative Control	UUCUCCGAACGUGUCACGUTT	ACGUGACACGUUCGGAGAATT
Beclin-1 (1)	GUGGAAUGGAAUGAGAUUATT	UAAUCUCAUUCCAUUCCACTT
Beclin-1 (2)	GCUGCCGUUAUACUGUUCUTT	AGAACAGUAUAACGGCAGCTT
FIP200 (1)	GGAGGACUGUUCAAAUUCATT	UGAAUUUGAACAGUCCUCCTT
FIP200 (2)	GCAGGAUACAAAUGUAUGUTT	ACAUACAUUUGUAUCCUGCTT
RhoA (1)	CAGCCCUGAUAGUUUAGAATT	UUCUAAACUAUCAGGGCUGTT
RhoA (2)	CCAGAAGUCAAGCAUUUCUTT	AGAAAUGCUUGACUUCUGGTT
Rubicon (1)	GGACUGACCCUGAUUACAUTT	AUGUAAUCAGGGUCAGUCCTT
Rubicon (2)	GGGUCCAGCUGUGUCACAUTT	AUGUGACACAGCUGGACCCTT

Primers for genotyping PCR	Mutant Forward	Common	Wild type Forward
Beclin-1	CTCCAGACTGCCTTGGGAAAA	TGGAAACAGGGTCTCATTCA	AGCCTCTGAAACTGGACACG
