# Supplementary material for: A mosquito salivary protein promotes flavivirus transmission by activation of autophagy
Source: Nat Commun. 2020 Jan 14;11:260. doi: 10.1038/s41467-019-14115-z (PMC6959235; doi:10.1038/s41467-019-14115-z)
Supplement: Supplementary file 2 — Reporting Summary [file 41467_2019_14115_MOESM2_ESM.pdf]

nature research

Corresponding author(s): Gong Cheng

Last updated by author(s): Nov 24, 2019

Reporting Summary

Nature Research wishes to improve the reproducibility of the work that we publish. This form provides structure for consistency and transparency in reporting. For further information on Nature Research policies, seeAuthors & Referees and theEditorial Policy Checklist .

Statistics

For all statistical analyses, confirm that the following items are present in the figure legend, table legend, main text, or Methods section.

n/a	Confirmed	
☐	☒	The exact sample size (*n*) for each experimental group/condition, given as a discrete number and unit of measurement
☐	☒	A statement on whether measurements were taken from distinct samples or whether the same sample was measured repeatedly
☐	☒	The statistical test(s) used AND whether they are one- or two-sided<br>*Only common tests should be described solely by name; describe more complex techniques in the Methods section.*
☐	☒	A description of all covariates tested
☐	☒	A description of any assumptions or corrections, such as tests of normality and adjustment for multiple comparisons
☐	☒	A full description of the statistical parameters including central tendency (e.g. means) or other basic estimates (e.g. regression coefficient) AND variation (e.g. standard deviation) or associated estimates of uncertainty (e.g. confidence intervals)
☐	☒	For null hypothesis testing, the test statistic (e.g. *F*, *t*, *r*) with confidence intervals, effect sizes, degrees of freedom and *P* value noted<br>*Give P values as exact values whenever suitable.*
☒	☐	For Bayesian analysis, information on the choice of priors and Markov chain Monte Carlo settings
☒	☐	For hierarchical and complex designs, identification of the appropriate level for tests and full reporting of outcomes
☒	☐	Estimates of effect sizes (e.g. Cohen's *d*, Pearson's *r*), indicating how they were calculated

Our web collection on statistics for biologists contains articles on many of the points above.

Software and code

Policy information about availability of computer code

Data collection	Zeiss LSM 780 confocal microscopy for image acquisition and data analysis.
Data analysis	Statistical analyses were performed using Graphpad Prism 6 software. Zeiss LSM 780 confocal microscopy for image acquisition and data analysis. Quantification data was analyzed by ImageJ 1.42q

For manuscripts utilizing custom algorithms or software that are central to the research but not yet described in published literature, software must be made available to editors/reviewers. We strongly encourage code deposition in a community repository (e.g. GitHub). See the Nature Research guidelines for submitting code & software for further information.

Data

Policy information about availability of data

All manuscripts must include a data availability statement. This statement should provide the following information, where applicable:

- Accession codes, unique identifiers, or web links for publicly available datasets
- A list of figures that have associated raw data
- A description of any restrictions on data availability

All data generated or analyzed during this study are included in this manuscripts and its supplementary information files.

Field-specific reporting

Please select the one below that is the best fit for your research. If you are not sure, read the appropriate sections before making your selection.

☒ Life sciences ☐ Behavioural & social sciences ☐ Ecological, evolutionary & environmental sciences

Life sciences study design

All studies must disclose on these points even when the disclosure is negative.

Sample size	No statistical method were used to pre-determine the sample size. Samples sizes were selected based on previous experience to obtain statistical significance and reproducibility.
Data exclusions	The mosquitoes that died before measurement were excluded from the analysis. The criteria was pre-established.
Replication	Experimental findings were reliably reproduced in multiple independent experiments as indicated throughout the manuscript.
Randomization	All animals in our experiments were randomly allocated into different groups.
Blinding	The investigators were not blinded to the allocation during the experiments or to the outcome assessment. The data presented did not require the use of blinding.

Reporting for specific materials, systems and methods

We require information from authors about some types of materials, experimental systems and methods used in many studies. Here, indicate whether each material, system or method listed is relevant to your study. If you are not sure if a list item applies to your research, read the appropriate section before selecting a response.

Materials & experimental systems

n/a	Involved in the study	
☐	☒	Antibodies
☐	☒	Eukaryotic cell lines
☒	☐	Palaeontology
☐	☒	Animals and other organisms
☐	☒	Human research participants
☒	☐	Clinical data

Methods

n/a	Involved in the study	
☒	☐	ChIP-seq
☒	☐	Flow cytometry
☒	☐	MRI-based neuroimaging

Antibodies

Antibodies used	For anti-AaVA-1 polyclonal antibody generation, recombinant AaVA-1 protein was expressed in the Escherichia coli BL21 DE3 strain in an insoluble form in inclusion bodies. The protein was then solubilized in 8 M urea and purified with a TALON Purification Kit (Cat# 635515, Clontech). Mouse antiserum was produced via inoculation with recombinant AaVA-1 with three boosts. Anti-LC3B (Cat# 2775), anti-Clathrin (Cat# 4796), anti-Caveolin-1 (Cat# 3267), anti-Flotillin-1 (Cat# 18634), anti-Rab7 (Cat# 9367), anti-Rab5 (Cat# 2143), anti-Lamp1 (Cat# 9091), anti-GM130 (Cat# 12480), anti-Tom20 (Cat# 42406), anti-Calnexin (Cat# 2679), anti-FIP200 (Cat# 12436) and anti-GAPDH (Cat# 2118) antibodies were obtained from Cell Signaling Technology (CST). Anti-Beclin-1 (Cat# 66665-1-Ig), anti-RhoA (Cat# 10749-1-AP), anti-Rubicon (Cat# 21444-1-AP), anti-Actin (Cat# 60008-1-Ig) and anti-LRPPRC (Cat# 21175-1-AP) antibodies were obtained from the Proteintech Group. Anti-Cdc42 (Cat# ab64533) antibody was obtained from Abcam. Anti-Arf6 (Cat# PA1-093), anti-V5-HRP (Cat# R961-25) and anti-Myc-HRP (Cat# R951-25) antibodies were purchased from Invitrogen. Anti-V5 (Cat# M167-3) and anti-Myc (Cat# 562) antibodies were purchased from Medical & Biological Lab (MBL). Anti-LRPPRC (Cat# sc166178, Santa Cruz) and anti-Beclin-1 (Cat# AF5295, R&D Systems) were only used for the staining of localization of LRPPRC and Bcelin-1/AaVA-1 including mitochondria. Anti-dsRNA J2 (Cat# 10010200) was obtained from English and Scientific Consulting Kft (SCICONS).
Validation	The antibody validation of immunoblot provided on supplier website using multiple murine and human cell lysates.

Eukaryotic cell lines

Policy information about cell lines

Cell line source(s)	The Vero, 293T, C6/36 and THP-1 cell lines were purchased from the ATCC (Cat# CCL81, CRL-3216, CRL-1660 and TIB-202). The Drosophila S2 cell line (Cat# R690-07) and FreeStyle-293F Cells (Cat# R790-07) were provided by Invitrogen.
Authentication	They have been authenticated using STR by ATCC and Invitrogen.
Mycoplasma contamination	They were negative for mycoplasma contamination.
Commonly misidentified lines (See ICLAC register)	No commonly misidentified cell line was used.

Animals and other organisms

Policy information about studies involving animals; ARRIVE guidelines recommended for reporting animal research

Laboratory animals	AG6 mice were donated by the Institute Pasteur of Shanghai, Chinese Academy of Sciences. Beclin-1+/– mice (Cat# 018429) were purchased from the Jackson Laboratory. Groups of age- and sex-matched mice, 8-10 weeks of age, were used for the mice studies. A. aegypti (the Rockefeller strain) and A. albopictus (the Jiangsu strain) were used for mosquito experiments
Wild animals	The study did not involved wild animals.
Field-collected samples	The study did not involved field-collected samples.
Ethics oversight	All animal protocols used in this study were approved by the Institutional Animal Care and Use Committee (IACUC) of Tsinghua University and performed in accordance with their guidelines.

Note that full information on the approval of the study protocol must also be provided in the manuscript.

Human research participants

Policy information about studies involving human research participants

Population characteristics	Healthy donor, 20-30 years of age, male and female
Recruitment	Human blood was collected from healthy donors randomly who provided written informed consent.
Ethics oversight	The protocol was approved by the local ethics committee of Tsinghua University.

Note that full information on the approval of the study protocol must also be provided in the manuscript.
